# The driving factors of corporate carbon emissions: an application of the LASSO model with survey data

**DOI:** 10.1007/s11356-023-26081-7

**Published:** 2023-03-15

**Authors:** Mengyao Xia, Helen Huifen Cai

**Affiliations:** 1 School of Management Engineering, Nanjing University of Information Science & Technology, Nanjing, 210044 Jiangsu Province China; 2 Business School, Middlesex University London, London, NW4 2BT UK

**Keywords:** Corporate carbon emissions, Determinants, LASSO regression model, Fixed-effect model

## Abstract

Corporate carbon performance is a key driver of achieving corporate sustainability. The identification of factors that influence corporate carbon emissions is fundamental to promoting carbon performance. Based on the carbon disclosure project (CDP) database, we integrate the least absolute shrinkage and selection operator (LASSO) regression model and the fixed effects model to identify the determinants of carbon emissions. Furthermore, we rank determining factors according to their importance. We find that *Capx* enters the models under all carbon contexts. For *Scope 1* and *Scope* 2, financial-level factors play a greater role. For *Scope 3*, corporate internal incentive policies and emission reduction behaviors are important. Different from absolute carbon emissions, for relative carbon emissions, the financial-level factors’ debt-paying ability is a vital reference indicator for the impact of corporate carbon emissions.

## Introduction

Reducing greenhouse gas (GHG) emissions is now the target of global efforts, as increased carbon emissions are the main cause of environmental deterioration. Under this context, how to decrease carbon emissions has become a topic of research that is incredibly significant at both the international and domestic levels. To achieve the emission reduction target, scholars began to study the factors affecting carbon emissions (Jiang et al. 2021). Druckman and Jackson (2016) find that household consumption accounts for about 72% of global carbon emissions. Thus, they studied the drivers of carbon emissions at the household level. Lamb et al. (2014) and Karasoy (2019) explore the driving factors affecting carbon emissions at the national level. Azizalrahman and Hasyimi (2019) dissect urbanization into sectors: residential, commercial, and industrial to explore urban sector drivers of carbon emissions. Li et al. (2018) use the structural decomposition analysis to uncover the driving forces of urban CO_2_ emission change in China. The existing research analyzes the drivers of carbon reduction at the national, city, and household levels from a macroperspective. As one of the main carriers affecting global warming, corporations improving the performance of carbon emissions can effectively alleviate environmental stress. However, few studies explore the factors that affect carbon emissions at the micro-firm level.

Prior studies have found that the impact of factors such as corporate inherent characteristics, the external environment, and corporate climate strategy behavior on carbon emission reduction has mixed results. Firm size (Lee 2012), political connection (Jiang et al. 2021), the carbon reporting decision (Córdova et al. 2018), industry category, sustainability reporting (Córdova et al. 2018), existence of a sustainability committee (Córdova et al. 2018), international experience of CEO and board of directors (Amran et al. 2016), organizational slack (Amran et al. 2016), emission trading policy (Chen et al. 2018), and social culture (Liu et al. 2018) have significant positive impacts on carbon emission reduction, whereas countries of the firm headquarters (Córdova et al. 2018), state ownership (Yang et al. 2019), and energy prices (Chen et al. 2018) have significant negative impacts on carbon emission reduction. Most previous studies have used panel regression models, which cannot shed light on the relative importance of impact factors. Thus, it is necessary to choose LASSO regression models that can be prioritized to explore the determinants of corporate carbon emissions.

In recent years, a series of legally binding climate change treaties, such as the United Nations Framework Convention on Climate Change (UNFCCC), the Kyoto Protocol, and the Paris Agreement, have been developed internationally to better assume environmental responsibility and jointly tackle climate change. However, as a major world power in the USA, the attitude toward acceding to international treaties is vague because of greater emission responsibilities and economic burdens. Matsumura et al. (2014) argue that firms may be penalized by capital markets for higher emission levels, leading to a decreased firm value. Thus, exploring the relationship between corporate costs of reducing emissions and financial performance is critical for improving the environment, achieving corporate sustainability, and allowing policymakers to mitigate carbon emissions.

This study makes the following contributions to the extant literature. Firstly, in terms of methods, we introduce the LASSO regression model to investigate the driving factors influencing corporate carbon emissions. LASSO provides an objective and comprehensive data-driven approach to capture the most important drivers of corporate carbon emissions. Secondly, we extend the findings of Jiang et al. (2021) to broaden the range of drivers that impact carbon emission reduction. Jiang et al.’s (2021) paper only discusses the driving factors of corporate emission reduction from the five aspects of political ties, corporate scale, industry category, regional disparity, and environmental regulation. We use the LASSO regression model to contain more internal and external factors. Compared with existing research which only explores the positive and negative impacts of driving factors on carbon emission reduction, LASSO regression is not restricted to the verification of whether each variable exerts an impact on corporate carbon emissions but to decide the priority of the driving factors and ranking them. This affords policymakers more flexibility in determining policy interventions, not only provide both a more accurate quantitative basis for policymakers and a theoretical basis, but also make contributions to the existing literature and corporate decision-making. Thirdly, we combine corporate carbon performance and financial performance indicators and discuss the importance of the impacting factors from the perspective of corporate environmental responsibility and profit development, which supplements the literature on corporate performance and provides theoretical guidance for managers to achieve corporate performance.

The rest of the paper is organized as follows: the “Literature review” section provides the literature review, the “Methodology and data” section describes the methodology and the data collection, the “Results” section shows the results, and the “Discussion and policy implications” section presents the discussion, policy implications, and future research directions.

## Literature review

**Fig. 1:**
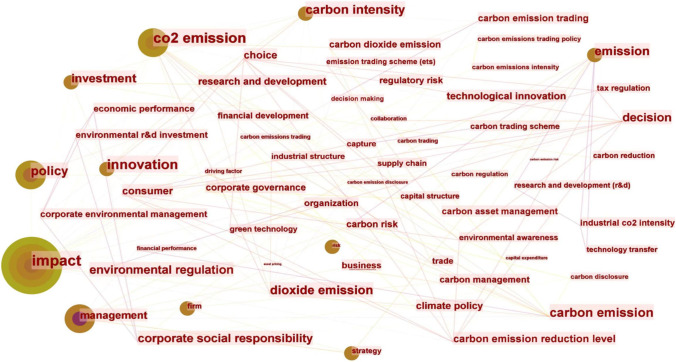
A visualization of the impact factors in corporate carbon emissions

With the intensification of global warming, carbon emissions have become a key concern for corporations. Countries are beginning to work together to reduce GHG emissions, and indeed, this has become a required goal for corporations in terms of environmental performance. However, the pursuit of corporate environmental performance has a mixed impact on corporate development (Dixon-Fowler et al. 2013). Earlier scholars put forward two markedly different views. The traditional economic trade-off argument posits that corporations incur large costs to improve environmental performance, and these additional financial burdens reduce corporate profits and value (Walley and Whitehead 1994). In contrast, the revisionist view argues that corporations can improve their economic performance by exploiting environmental opportunities as a first mover (Esty and Porter, 1998; Reinhardt, 1999). A visualization of the impact factors in corporate carbon emissions is shown in Fig. 1. Corporate carbon emissions are affected by a combination of factors, which we divide into three categories.

Firm-level factors are one category that affects corporate carbon emissions. If corporations are to address climate change, it cannot be viewed as an isolated environmental issue. It is important to integrate climate change into corporate business strategies (Amran et al. 2016). McKinsey (2008) found that more than 30% of the executives admitted to seldom or never including climate change in business strategies. In corporate emission reduction strategies, executive (especially CEO) attitudes and characteristics play a very important role, such as CEO compensation, CEO power, and CEO duality (Raghunandan and Rajgopal 2022; Hossain et al. 2022). Under the complex operating activities, not only the CEO but also the board of directors plays an important role in corporate emission reduction. The relationship between board characteristics and carbon emissions, such as foreign directors, board gender diversity, outside directors, and the number of directors, is widely studied by scholars (Mardini and Lahyani 2021; Nuber and Velte 2021; Kurnia et al. 2020). Liao et al. (2015) argue that independent directors are more willing to pursue environmental opportunities to acquire more reputation and honor. Nuber and Velte (2021) find that women directors exhibit a strong orientation toward environmental responsibility and are more concerned with environmental issues. Mardini and Lahyani (2021) find that foreign directors are more engaged in sustainability and influence the board’s decisions toward supporting climate change activities. These board characteristics all have positive impacts on decreasing carbon emissions. Corporations use different reporting boundaries and accounting methodologies when calculating amounts of carbon emissions (Stanny 2018). If energy expenses relative to total expenses are higher, the corporations invest more in environmental energy projects and so achieve lower emissions (Mahapatra et al. 2021).

Carbon action–level factors are a category that affects corporate carbon emissions. Good corporate awareness of environmental issues promotes pro-environmental activities (Sharma 2000). Awareness of the environment can be divided into carbon-risk awareness and carbon opportunity awareness. Compared to carbon opportunity awareness, corporations with a greater awareness of carbon risk not only exhibit a greater willingness to develop mutually beneficial relationships with stakeholders to enhance corporate capacity to generate sustainable development but also will adopt a variety of governance mechanisms to promote corporate emission reduction, such as setting carbon targets, providing carbon reduction incentives, and linking compensation to carbon reduction (Luo and Tang 2021; Jung et al. 2018). Researchers find that incentives are adopted by firms to reduce carbon emissions from their operations. Eccles et al. (2012) argue that monetary incentives lead to higher carbon emissions, while non-monetary incentives lead to lower carbon emissions. A growing number of global initiatives are supporting corporate non-financial target-setting efforts. Different types of corporate climate change targets exhibit different behaviors regarding trading corporate carbon. Compared to absolute targets, intensity targets reflect ambitions to reduce GHG emissions at a more relative level (Slawinski et al. 2017; Dahlmann et al. 2019). To achieve lower carbon emissions, corporations participate in the carbon emission trading system (ETS) to achieve carbon credit purchases, implement internal carbon pricing (ICP) mechanisms within corporations, and actively promote investment in emission reduction activities. Firms find that voluntarily reducing carbon emissions often brings economic benefits (Hart 1997).

Financial-level factors are a category that affects corporate carbon emissions. The relationship between corporate environmental performance and profitability is extensively studied in the existing literature (Larasati et al. 2020; Dixon-Fowler et al. 2013; Guenther and Hoppe 2014). R&D is often considered a financial-level impact factor. Under regulatory pressure from carbon emissions, corporations are trying to “offset” the additional costs of regulatory compliance through innovation. As an effective means to promote corporation innovation, R&D can effectively affect corporate carbon emissions (Lanoie et al. 2011). Corporate capital expenditures are associated with a larger carbon footprint and will lead to more carbon emissions (Karim et al. 2021). Trade-off theory suggests that firms with a high leverage ratio have higher carbon emissions (Andreoni and Galmarini 2012). There is a negative relationship between market-to-book ratios and carbon emissions because the carbon premium is unlikely to be driven by cash flow effects related to productivity (Bolton et al. 2022).

## Methodology and data

### LASSO regression model

**Fig. 2:**
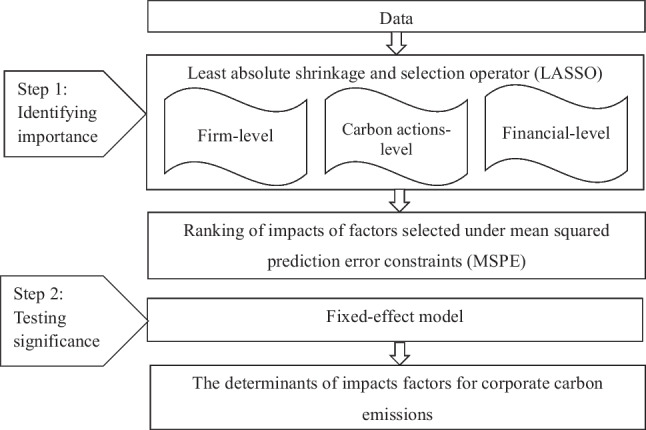
Research framework

We chose to integrate the LASSO and the fixed effects model into identifying determinants of corporate carbon emissions. Firstly, we take the absolute carbon emissions of total, Scope 1, Scope 2, and Scope 3 and the relative carbon emissions of per revenues and per full-time equivalent employees as dependent variables, respectively. Then, we applied the LASSO regression model to rank the importance of factors affecting carbon emissions and capture the important preferences of influencing factors on the different corporate carbon emission scopes through the fixed effects model. By integration of the models, we can consider the factors affecting carbon emissions from more dimensions. Figure 2 shows the framework of our methodology.

Proposed by Tibshirani (1996), LASSO is a regression variable selection method that automates model selection. As a selection procedure, it combines the least squares method with a constraint on the sum of the absolute values of the coefficients to improve prediction accuracy and interpretability. Considering ordinary linear models, supposing ${y}_{i}={\left({y}_{1},{y}_{2},{y}_{3},\dots ,{y}_{d}\right)}^{T}$ is the response variable and $X=\left({X}^{\left(1\right)},{X}^{\left(2\right)},{X}^{\left(3\right)},\dots ,{X}^{\left(4\right)}\right)$ is the covariate for each ${X}^{\left(j\right)}={\left({X}_{1}^{\left(j\right)},{X}_{3}^{\left(j\right)},{X}_{3}^{\left(j\right)},\dots ,{X}_{d}^{\left(j\right)}\right)}^{T}$, *β* = (*β*_*1*_,* β*_*2*_,* β*_*3*_,*…*,* β*_*1*_*,*):

1 $${Y}_{i}={X}_{i}^{T}\beta +{\varepsilon }_{i}$$

where *ε*_*i*_ is an error term.

When *X* is a full rank design matrix, the regression coefficient *β* can be obtained by the ordinary least squares estimation method:

2 $${\widehat{\beta }}_{\mathrm{OLS}}=\mathrm{arg}\underset{\beta \in {R}^{d}}{\mathrm{min}}{\Vert {Y}_{i}-X\beta \Vert }^{2}={\left({X}^{T}X\right)}^{-1}{X}^{T}{Y}_{i}$$

where *d* is the number of the covariates.

When the design matrix *X* does not meet the full rank, the penalty method is introduced to achieve the effect of variable selection by compressing some parameters to zero. The penalty method is to take the minimum value of the penalty likelihood function as the estimated value of the regression coefficient; this is shown below:

3 $$\widehat{\beta }=\mathrm{arg}\underset{\beta \in {R}^{d}}{\mathrm{ min}}{\Vert {Y}_{i}-X\beta \Vert }^{2}={P}_{\lambda }\left(\left|\beta \right|\right)$$

where ${P}_{\lambda }\left(\left|\beta \right|\right)=\lambda \sum \limits_{j=1}^{d}{\left|{\beta }_{j}\right|}^{m},m\ge 0$ is the penalty term (is also named as the tuning parameter). When *m* = 1, $\lambda \sum \limits_{j=1}^{d}\left|{\beta }_{j}\right|$ is the *L*_1_ norm of the parameter vector. λ is a nonnegative regularization parameter. *β*_*j*_ are the other parameters.

By adding the *L*_1_ norm to the ordinary linear model, the LASSO estimate is shown below:

4 $$\begin{array}{c}{\widehat{\beta }}_{L\mathrm{asso}}=\mathrm{arg}\underset{\beta \in {R}^{d}}{\mathrm{ min}}\frac{1}{N}{\Vert {Y}_{i}-X\beta \Vert }_{2}^{2}\\ s.t. \sum \limits_{j=1}^{d}\left|\beta \right|\le t,t\ge 0\end{array}$$

where *t* ≥ 0 is a pre-specified free parameter that is chosen to determine the amount of regularization through cross-validation. ${t}_{0}\sum \limits_{j=1}^{d}\left|{\widehat{\beta }}_{j}\left(\mathrm{OLS}\right)\right|$, when *t* < *t*_0_, a part of the coefficient will be compressed to zero, thereby reducing the dimension of *X* and reducing the complexity of the model. *N* is the total number of observations.

The LASSO estimator $\widehat{\beta }$ can be equivalently written in Lagrangian form as

5 $${\widehat{\beta }}_{L\mathrm{asso}}=\mathrm{arg}\underset{\beta \in {R}^{d}}{\mathrm{ min}}\left(\frac{1}{N}{\Vert {Y}_{i}-X\beta \Vert }_{2}^{2}+\lambda \sum_{j=1}^{d}\left|\beta \right|\right)$$

where *t* corresponds to *λ* one-to-one and is the adjustment coefficient. *λ* is the regularization parameter and the higher the value of *λ*, the lower the number of non-zero *β* and vice versa.

According to the above equations, we can derive a sparse regression model which regularizes the parameters *β* under sparse assumption. When *λ* is exceptionally large, the value of all the parameters of the independent variables is zero. By adjusting the value of *λ*, the parameters will gradually increase and turn from zero to non-zero one by one. Then, based on the sequence of the appearance of the parameters, the degree of importance of the different independent variables can be known for prediction.

We introduced *K*-fold cross-validation to estimate the best regularization parameters $\lambda$ or *t*. Firstly, the data set was randomly split into *K* approximately equal-sized sets. The first subsample was left as the “validation set” and the remaining *K*-1 subsamples were used as the “training set” to estimate the model. We then predicted the first subsample and calculated the mean squared prediction error (MSPE) for the first subsample. Secondly, the second subsample was used as the validation set, while the remaining *K*-1 subsamples were used as the training set to predict the second subsample and calculate the MSPE of the second subsample. By analogy, we performed *k* training runs in turn in *K* sets for validation. Then, we added up the MSPE of all the sub-samples and took the average test error over the *K* runs, which was regarded as the test error for the regression model. Finally, the regularization parameters *λ* were selected so that they corresponded to the lowest estimated generalization error, which consequently gives the best predictive power.

To estimate the regression coefficient vector *β*, we repeated it multiple times on different values of $\lambda$ (Shi et al. 2020). Specifically, the optimized *λ* was set for all coefficients except the intercept that was forced to zero and was computed according to a geometric sequence. We computed the largest *λ* and the smallest *λ*, while making the largest value of *λ* 10,000 times the smallest value. The 100 specifications sets of regressions were run with different values of *λ*, denoted as SP (Shum et al. 2021). Specification 1 and specification 100 are the specifications with the smallest value of *λ* and the largest value of *λ*. When the corresponding *λ* or SP values increase, the coefficient of an independent variable increases from zero. The first independent variable with a non-zero coefficient has the most influence on corporate carbon emissions. The earlier the variable appears, the more important it is for prediction. Thus, using multiple iterations of the LASSO method, we could observe changes in the importance of independent variables. To identify independent variables that are important enough, we selected the $\lambda$ value at the MSPE. Then, we included those variables in a fixed model to explore the significance of corporate carbon emission factors.

### Data

We identified a range of potential factors that affect corporate carbon emissions from both the Carbon Disclosure Project (CDP) database and the Compustat database. The CDP was established in 2000 as a non-governmental organization (NGO) in the UK. The CDP asks firms to describe climate change management strategies, to identify climate change and its risks and opportunities and to disclose GHG emissions. Many of the world’s largest firms responded to the CDP survey requests; by 2015, more than 5500 firms had responded (CDP, 2018). The BoardEx database has compiled the full list of their directors, senior managers, and disclosed moneymakers for over 18,000 corporations worldwide and has built complete profiles on each individual. Firm-level and carbon action–level information were obtained from the CDP database and BoardEx database in 2009–2019. Firm-level information included business strategy, GHG inventory boundary, individual positions, CEO duality, the number of directors serving on the board, energy consumption, energy consumption intensity, total compensation, nationality mix proportion, the proportion of male directors, and the number of directors. The carbon action–level includes carbon awareness, identity climate change risks, identity climate change opportunities, incentive for climate change issues, benefit from incentive, incentive type, emission reduction target, emission reduction activities, internal carbon price, emission reduction initiatives, third party, carbon credits, emission trading schemes, public policy, voluntarily published information, and value chain. Only CEO duality, the number of directors serving on the board, total compensation, nationality mix proportion, the proportion of male directors, and the number of directors were obtained from the BoardEx database; the others were all obtained from the CDP database. The Compustat database provides nearly 20 years of historical data on financial indicators for North American publicly traded corporations. Therefore, financial-level information was obtained from Compustat for 2009–2019. Such information included debt-paying ability, operation capability, profitability, growth ability, R&D, total assets turnover, capital expenditure, asset intensity, firm leverage, market-to-book ratio, debt-to-asset ratio, and return on assets. We employed unbalanced panel data estimate approaches and controlled for the year fixed effects in our model. After matching the data with the CDP database, the BoardEx database, and the Compustat database and deleting observations with missing values, we were left with 4013 observations. Tables 1 and 2 present the variable definitions and the descriptive statistics for our sample.

**Table 1:** Variable definitions

Variable	Definition	Source
*Total*	The sum of organization’s emissions of Scope 1, Scope 2, and Scope 3 in metric tons CO_2_e	CDP
*Scope 1*	Organization’s gross global Scope 1 emissions in metric tons CO_2_e	CDP
*Scope 2*	Organization’s gross global Scope 2 emissions in metric tons CO_2_e	CDP
*Scope 3*	Organization’s gross global Scope 3 emissions in metric tons CO_2_e	CDP
Carbon emissions per revenues (*Rin*)	Scope 1 plus Scope 2 carbon emissions/total operating revenue	CDP
Carbon emissions per full-time equivalent employees (*Ein*)	Scope 1 plus Scope 2 carbon emissions/equivalent employees	CDP
Firm level		
* Strategy*	Business strategy: 1 if climate-related issues integrated into business strategy, 0 otherwise	CDP
Boundary (*Operate*/*Finance*)	Greenhouse gas inventory boundary: (1) if the greenhouse gas inventory boundary is operation control (*Operate*); (2) if the greenhouse gas inventory boundary is financial control (*Finance*)	CDP
Manager (*CEO*/*Team*)	Individual positions who occupy the highest level of direct responsibility for climate change within organizations: (1) if the manager is a CEO (*CEO*); (2) if the manager is a team (*Team*)	CDP
CEO duality (*Founder*/*Dual*)	CEO duality: (1) if CEO as firm founder (*Founder*); (2) if CEO as chairman of the board (*Dual*)	BoardEx
* Boardamount*	The number of directors serving on the board	BoardEx
* Opexpense*	Energy consumption: 1 if corporation energy percentage of total operational spend, 0 otherwise	CDP
* Opexpense05*	Energy consumption intensity. Energy percentage of total operational spend more than 0% but less than or equal to 5%	CDP
* Opexpense510*	Energy consumption intensity. Energy percentage of total operational spend more than 5% but less than or equal to 10%	CDP
* Opexpense1015*	Energy consumption intensity. Energy percentage of total operational spend more than 10% but less than or equal to 15%	CDP
* TDC*	TDC is the total compensation for the fiscal year, including salary, bonus, total value of restricted stock and stock options granted, and long-term incentive payouts	BoardEx
* Nationalitymix*	Nationality mix proportion of Directors from different countries at the annual report date selected	BoardEx
* Genderratio*	The proportion of male directors at the annual report date selected	BoardEx
* Numberdirector*	Number of executive directors, supervisory directors or all of the directors at the annual report date selected	BoardEx
Carbon action level		
* Awareness*	Carbon awareness: 1 if corporations identify any climate change risks and/or opportunities, 0 otherwise	CDP
* Risk*	Identify climate change risks: 1 if corporations identify any climate change risks, 0 otherwise	CDP
* Oppo*	Identify climate change opportunities: 1 if corporations identify any climate change opportunities, 0 otherwise	CDP
* Incentive*	Incentives for climate change issues: 1 if corporations provide incentives for the management of climate change issues	CDP
Benefit from incentive (*Employees*/*Managerexe*)	Who is entitled to benefit from the incentives provided for the management of climate-related issues. (1) *Employees*; (2) *Managerexe*	CDP
* Monetary*	Incentive type. 1 if the incentive type is monetary, 0 otherwise	CDP
* Target*	Emissions reduction target: 1 if corporations have emissions reduction target, 0 otherwise	CDP
Types of targets (*Intensity*/*Absolute*)	Types of emissions reduction targets. (1) Intensity targets (*Intensity*); (2) Absolute targets (*Absolute*)	CDP
* Regulatory*	Emissions reduction activities: 1 if corporations use compliance with regulatory requirements/standards to drive investment in emissions reduction activities	CDP
* Incentiveemp*	Emissions reduction activities: 1 if corporations use employee engagement to drive investment in emissions reduction activities	CDP
* Energy*	Emissions reduction activities: 1 if corporations use dedicated budget for energy efficiency to drive investment in emissions reduction activities	CDP
* Icp*	Internal carbon price: 1 if corporations use internal price on carbon (*1cp*) to drive investment in emissions reduction activities, 0 otherwise	CDP
* Carbonmes*	Emissions reduction initiatives: 1 if corporations have emissions reduction initiatives, 0 otherwise	CDP
* Thirty*	Third party: 1 if the use of goods and/or services directly enable GHG emissions to be avoided by a third party, 0 otherwise	CDP
* Credit*	Carbon credits: 1 if corporations originated any project-based carbon credits or purchased, 0 otherwise	CDP
* Ets*	Emission trading schemes: 1 if corporations participate in any emission trading schemes, 0 otherwise	CDP
* Touch*	Public policy: 1 if directly or indirectly influence public policy on climate-related issues, 0 otherwise	CDP
* Voluntary*	Voluntary publish information: 1 if corporations publish information about organization’s response to climate change and GHG emissions performance in places other than in your CDP response, 0 otherwise	CDP
* Value*	Value chain. Do you engage with your value chain on climate-related issues?	CDP
Financial level		
* Currentratio*	Debt-paying ability. Current assets divided by current liabilities	Compustat
* Quickratio*	Debt-paying ability. Cash flow from operations divided by current liabilities	Compustat
* Netprosales*	Operation capability Net profit margin on sales. Net profit divided by proceeds of sale	Compustat
* Operprotio*	Operation capability. Operating profit ratio. Operating profit divided by operating revenue	Compustat
* Receiturntio*	Profitability. Receivables turnover ratio. Net income from main business divided by average balance of accounts receivable	Compustat
* Inventurn*	Profitability. Inventory turnover. Cost of main business divided by average inventory	Compustat
* Capitalstock*	Growth ability. The proportion of capital stock. Capital stock divided by total equity turnover	Compustat
* Totassgrate*	Growth ability. Total assets growth rate. Total assets at year-end divided by total assets at year-beginning	Compustat
* R&D*	Research and development investment	Compustat
* Totassover*	Total assets turnover. Operating income before depreciation is divided by total assets	Compustat
* Capex*	Capital expenditure. The total capital divided by total sales	Compustat
* Asset*	Asset intensity. Total assets divided by all employees	Compustat
* Leverage*	Firm leverage. Long-term debt plus current liabilities deflated by total assets	Compustat
* Mtbt*	Market-to-book ratio. The ratio of market-to-book value of equity	Compustat
* Lev*	Debt-to-asset ratio. Total assets divided by total liabilities	Compustat
* ROA*	Return of assets. Profit after taxes divided by total assets	Compustat

**Table 2:** Descriptive statistics

Variable	Observations	Mean	Std. dev	Min	Max
*Total*	3465	13.72	2.654	2.079	22.33
*Scope 1*	3539	11.62	3.002	− 2.226	18.89
*Scope 2*	3477	12.15	1.924	0	17.81
*Scope 3*	2806	12.53	3.146	0.058	22.33
*Rin*	3559	3.785	2.068	− 6.376	13.22
*Ein*	3539	9.930	2.255	− 0.248	18.98
*Strategy*	4013	0.890	0.313	0	1
*Operate*	3705	0.749	0.434	0	1
*Finance*	3705	0.156	0.363	0	1
*Team*	4013	0.399	0.490	0	1
*CEO*	4013	0.117	0.322	0	1
*Founder*	4013	0.026	0.158	0	1
*Dual*	4013	0.379	0.485	0	1
*Opexpense*	3705	0.870	0.337	0	1
*Boardamount*	3662	0.221	0.122	0.010	1.150
*Opexpense*	3705	0.621	0.485	0	1
*Opexpense05*	3705	0.109	0.311	0	1
*Opexpense510*	4013	0.0431	0.203	0	1
*Opexpense1015*	3599	11.10	9.042	0	280.6
*TDC*	3508	0.156	0.193	0	0.900
*Nationalitymix*	3534	0.793	0.099	0.375	1
*Genderratio*	3534	10.94	2.137	3	19
*Numberdirector*	4013	0.804	0.397	0	1
*Risk*	4013	0.730	0.444	0	1
*Oppo*	4013	0.672	0.470	0	1
*Incentive*	4013	0.768	0.422	0	1
*Employees*	4013	0.394	0.489	0	1
*Managerexe*	4013	0.540	0.498	0	1
*Monetary*	4013	0.597	0.491	0	1
*Target*	4013	0.763	0.426	0	1
*Intensity*	4013	0.455	0.498	0	1
*Absolute*	4013	0.464	0.499	0	1
*Regulatory*	3705	0.417	0.493	0	1
*Incentiveemp*	3705	0.457	0.498	0	1
*Energy*	3705	0.376	0.484	0	1
*Icp*	3705	0.093	0.290	0	1
*Carbonmes*	4013	0.903	0.296	0	1
*Thirty*	4013	0.620	0.485	0	1
*Credit*	4013	0.165	0.371	0	1
*Ets*	4013	0.310	0.463	0	1
*Touch*	4013	0.638	0.481	0	1
*Voluntary*	4013	0.653	0.476	0	1
*Value*	3070	0.829	0.377	0	1
*Currentratio*	3433	0.408	0.588	− 3.522	5.657
*Quickratio*	3371	0.611	0.571	− 12.25	7.692
*Netprosales*	3546	0.142	0.175	− 2.298	1.410
*Operprotio*	3678	0.418	0.223	− 2.509	0.975
*Receiturntio*	3642	0.013	0.060	0	2.965
*Inventurn*	3157	0.165	0.557	0.001	20.94
*Capitalstock*	3994	0.009	0.206	− 4.114	11.26
*Totassgrate*	3989	− 1.378	25.80	− 1406	1
*R&D*	4013	2.763	3.096	0	10.17
*Totassover*	3540	− 2.519	0.775	− 8.367	− 0.461
*Capex*	3562	6.022	1.708	− 6.908	10.54
*Asset*	3967	6.731	1.451	1.517	13.83
*Leverage*	3888	− 1.452	0.891	− 9.426	1.607
*Mtbt*	3355	6.822	1.604	− 2.872	13.84
*Lev*	3994	− 0.496	0.434	− 5.480	2.275
*ROA*	3297	− 2.512	0.810	− 11.03	− 0.111

*Total*, *Scope 1*, *Scope 2*, *Scope 3*, *Rin*, and *Ein* are the dependent variables and as the proxy variable of corporate carbon emissions. See Table 1 for the definition of variables

## Results

### The regression of total impact factors to carbon emissions

Table 3 reports the regression of whole impact factors to carbon emissions. From columns (1) to (6), we report the regression results of carbon emissions in different measurement methods. In columns (1), (2), (3) and (4), we report the regression of absolute carbon emissions. In columns (5) and (6), we report the regression of relative carbon emissions. All the models are controlled for the year fixed effects. Firstly, we analyzed the effects of firm-level information on corporate carbon emissions. *Operate* has a positive effect on the corporate carbon emissions of* Scope 1*, *Rin*, and *Ein*. Greenhouse gas, measured in both operate control and financial control, has a positive impact on the reduction of corporate carbon emissions. The aggregate effect of *Scope 1*, *Rin*, and *Ein* decreases by 6.3%, 13%, and 9.5% respectively for a one-standard-deviation increase in *Operate*. The aggregate effects of *Scope 1*, *Scope 3*, *Rin*, and *Ein* decrease by 4.5%, 9.4%, 8.5%, and 6.7% respectively for a one-standard-deviation increase in *Finance*. The operate boundary has a greater impact on decreasing carbon emissions than that of the finance boundary. When the CEO is also the founder of firms, it contributes to reducing the carbon emissions of *Scope 1* and *Scope 2* by 2.8% and 5.3% respectively for a one-standard-deviation increase in *CEO*. However, *Dual* is not conducive to the reduction of corporate carbon emissions. Corporations should moderately decrease CEO’s discretion to ensure better implementation of emission reduction strategies. *Boardamount* can decrease 3.7% of the aggregate effect for both *Rin* and *Ein*, but it will increase the aggregate effect of *Scope 1* by 2.3% for a one-standard-deviation increase in *Boardamount.* Energy consumption intensity of *Opexpense05*, *Opexpense510*, and *Opexpense1015* has a positive influence on reducing carbon emissions. *Opexpense05* decreases the aggregate effect of *Total*,* Scope 1*, *Rin*, and *Ein* by 4.9%, 11.8%, 18.7%, and 14.9% respectively for a one-standard-deviation increase. *Opexpense510* decreases the aggregate effect of* Scope 1*, *Rin*, and *Ein* by 2.5%, 4.4%, and 3.1% respectively for a one-standard-deviation increase. *Opexpense1015* decreases the aggregate effect of *Rin* and *Ein* by 155.5% and 154.1% respectively for a one-standard-deviation increase. For *Scope 2* and *Scope 3*, the higher energy consumption intensity is less conducive to reducing carbon emissions. Although energy consumption intensity can decrease corporate carbon emissions, the role of *Opexpense* is not efficient. The corporations with energy consumption cannot be effective for emission reduction, which is insignificant because of energy consumption intensity that is too high. *TDC* decreases the aggregate effect of *Total*,* Scope 1*, *Scope 3*, *Rin*, and *Ein* by 0.1% for a one-standard-deviation increase. *Genderratio* has an efficiency on *Scope 3*, which decreases the aggregate effect of *Scope 3* by 135.1%. *Numberdirectors* has an efficiency on relative carbon emissions, which decreases the aggregate effect of both *Rin* and *Ein* by 0.6%. *Strategy* and *Nationalitymix* are insignificant on corporate carbon emissions.

**Table 3:** The regression of impact factors to carbon emissions

	(1)	(2)	(3)	(4)	(5)	(6)
	*Total*	*Scope 1*	*Scope 2*	*Scope 3*	*Rin*	*Ein*
*Strategy*	0.002	− 0.001	− 0.054	0.966**	− 0.148	− 0.228
	(0.177)	(0.185)	(0.148)	(0.384)	(0.153)	(0.146)
*Operate*	− 0.074	− 0.436***	0.089	− 0.433	− 0.622***	− 0.491***
	(0.159)	(0.160)	(0.130)	(0.321)	(0.132)	(0.126)
*Finance*	− 0.256	− 0.374**	0.277*	− 0.823**	− 0.533***	− 0.417***
	(0.175)	(0.177)	(0.143)	(0.348)	(0.146)	(0.140)
*Team*	0.067	0.394***	− 0.041	0.111	0.217***	0.229***
	(0.084)	(0.085)	(0.068)	(0.157)	(0.070)	(0.067)
*CEO*	0.148	0.405***	− 0.133	− 0.063	0.199**	0.175*
	(0.119)	(0.120)	(0.097)	(0.217)	(0.100)	(0.095)
*Founder*	− 0.064	− 0.541**	− 0.645***	0.342	0.052	− 0.001
	(0.253)	(0.257)	(0.204)	(0.453)	(0.213)	(0.204)
*Dual*	0.244***	0.456***	0.108*	0.363**	0.156**	0.136**
	(0.077)	(0.078)	(0.063)	(0.145)	(0.065)	(0.062)
*Boardamount*	0.559***	− 0.093	− 0.069	0.770	− 0.624*	− 0.691**
	(0.171)	(0.399)	(0.320)	(0.721)	(0.331)	(0.316)
*Opexpense*	− 0.170	0.697***	− 0.189	− 0.152	0.898***	0.748***
	(0.391)	(0.173)	(0.142)	(0.376)	(0.143)	(0.137)
*Opexpense05*	− 0.416***	− 1.138***	0.128	0.625***	− 1.241***	− 1.084***
	(0.125)	(0.126)	(0.101)	(0.237)	(0.105)	(0.100)
*Opexpense510*	− 0.105	− 0.364**	0.476***	0.837***	− 0.450***	− 0.342***
	(0.143)	(0.146)	(0.117)	(0.274)	(0.121)	(0.116)
*Opexpense1015*	− 0.071	− 0.136	0.105	0.228	− 0.356**	− 0.384***
	(0.177)	(0.183)	(0.146)	(0.322)	(0.152)	(0.145)
*TDC*	− 0.016***	− 0.020***	− 0.001	− 0.016**	− 0.015***	− 0.015***
	(0.004)	(0.004)	(0.003)	(0.007)	(0.003)	(0.003)
*Nationalitymix*	0.349*	0.324	0.368**	0.811**	0.241	0.344**
	(0.206)	(0.210)	(0.169)	(0.385)	(0.174)	(0.167)
*Genderratio*	− 0.553	0.065	0.101	− 1.988**	0.571	0.582*
	(0.422)	(0.421)	(0.342)	(0.828)	(0.350)	(0.334)
*Numberdirectors*	0.027	0.052**	− 0.005	0.057	− 0.032*	− 0.036**
	(0.022)	(0.022)	(0.018)	(0.042)	(0.019)	(0.02)
*Awareness*	− 0.027	− 0.027	0.163	0.259	− 0.015	0.101
	(0.228)	(0.227)	(0.182)	(0.450)	(0.189)	(0.180)
*Risk*	0.516***	0.732***	0.078	− 0.061	0.499***	0.435***
	(0.176)	(0.181)	(0.144)	(0.352)	(0.150)	(0.143)
*Oppo*	− 0.103	− 0.068	− 0.199**	− 0.058	− 0.118	− 0.134
	(0.119)	(0.116)	(0.093)	(0.224)	(0.096)	(0.092)
*Incentive*	0.006	0.452***	0.271**	0.148	0.200	0.209*
	(0.156)	(0.158)	(0.129)	(0.326)	(0.131)	(0.125)
*Employees*	0.255***	− 0.062	0.124*	0.438***	0.079	0.073
	(0.092)	(0.092)	(0.074)	(0.169)	(0.076)	(0.073)
*Managerexe*	0.209**	− 0.310***	0.244***	0.326*	− 0.103	− 0.074
	(0.103)	(0.104)	(0.084)	(0.197)	(0.087)	(0.083)
*Monetary: (baseline group: non-monetary reward)*	0.044	− 0.132	− 0.061	− 0.027	− 0.005	0.014
	(0.106)	(0.108)	(0.087)	(0.201)	(0.090)	(0.086)
*Target*	0.033	0.175	− 0.099	− 0.527*	0.296**	0.161
	(0.165)	(0.167)	(0.134)	(0.320)	(0.138)	(0.132)
*Intensity*	0.286**	0.107	− 0.057	0.628***	0.097	0.128
	(0.114)	(0.115)	(0.092)	(0.203)	(0.096)	(0.091)
*Absolute*	0.189*	− 0.234**	0.001	0.731***	− 0.197**	− 0.193**
	(0.111)	(0.112)	(0.090)	(0.203)	(0.093)	(0.089)
*Regulatory*	0.190**	0.200**	− 0.151**	0.368**	0.091	0.086
	(0.082)	(0.082)	(0.067)	(0.153)	(0.069)	(0.066)
*Incentiveemp*	− 0.176**	− 0.282***	0.164**	− 0.093	− 0.224***	− 0.247***
	(0.085)	(0.086)	(0.069)	(0.160)	(0.071)	(0.068)
*Energy*	0.197**	− 0.014	− 0.006	0.277*	0.117*	0.087
	(0.082)	(0.084)	(0.067)	(0.154)	(0.069)	(0.066)
*Icp*	0.560***	0.722***	− 0.147	0.256	0.801***	0.755***
	(0.134)	(0.136)	(0.110)	(0.238)	(0.113)	(0.108)
*Carbonmes*	0.398*	0.422*	0.434**	0.665	0.299*	0.382**
	(0.214)	(0.223)	(0.177)	(0.518)	(0.182)	(0.174)
*Thirty*	0.410***	0.158*	0.336***	0.965***	0.290***	0.233***
	(0.087)	(0.088)	(0.071)	(0.164)	(0.073)	(0.070)
*Credit*	− 0.001	0.092	− 0.040	0.467**	0.014	0.036
	(0.101)	(0.103)	(0.082)	(0.186)	(0.085)	(0.082)
*Ets*	0.659***	1.152***	0.545***	0.595***	0.634***	0.633***
	(0.089)	(0.090)	(0.073)	(0.166)	(0.075)	(0.071)
*Touch*	0.224**	0.237**	0.034	0.095	0.274***	0.280***
	(0.097)	(0.097)	(0.078)	(0.185)	(0.080)	(0.077)
*Voluntary*	− 0.240***	− 0.094	0.005	− 0.409**	− 0.035	− 0.003
	(0.085)	(0.085)	(0.068)	(0.165)	(0.070)	(0.067)
*Value*	0.307***	0.115	− 0.136	0.653**	0.019	0.056
	(0.118)	(0.121)	(0.098)	(0.257)	(0.100)	(0.096)
*Currentratio*	− 0.109	− 0.487***	0.513***	0.156	− 0.351***	− 0.1925**
	(0.099)	(0.098)	(0.078)	(0.185)	(0.081)	(0.078)
*Quickratio*	− 0.040	0.626***	− 0.142	− 0.646***	0.795***	0.725***
	(0.130)	(0.131)	(0.105)	(0.245)	(0.109)	(0.104)
*Netprosales*	1.510***	1.921***	0.474	2.933***	2.243***	0.257
	(0.564)	(0.569)	(0.459)	(1.048)	(0.473)	(0.452)
*Operprotio*	− 2.446***	− 3.061***	− 2.717***	− 2.252***	− 0.873***	− 1.827***
	(0.317)	(0.316)	(0.255)	(0.595)	(0.262)	(0.251)
*Receiturntio*	− 0.046	− 0.207	1.312**	− 2.222	− 0.396	0.335
	(0.825)	(0.805)	(0.640)	(1.408)	(0.669)	(0.640)
*Inventurn*	− 0.189***	− 0.173***	− 0.288***	− 0.099	− 0.158***	− 0.187***
	(0.061)	(0.065)	(0.051)	(0.105)	(0.054)	(0.051)
*Capitalstock*	− 1.673**	− 0.505	− 0.538	− 2.989**	0.906	0.661
	(0.715)	(0.727)	(0.578)	(1.230)	(0.604)	(0.578)
*Totassgrate*	0.072***	− 0.019	0.060***	0.100	0.040*	0.044**
	(0.025)	(0.029)	(0.021)	(0.066)	(0.021)	(0.021)
*R&D*	0.020	− 0.068***	0.058***	0.095***	− 0.066***	− 0.072***
	(0.016)	(0.017)	(0.013)	(0.030)	(0.014)	(0.013)
*Totassover*	− 0.017	0.186	− 0.015	− 0.003	− 0.225**	0.080
	(0.120)	(0.120)	(0.096)	(0.230)	(0.100)	(0.095)
*Capx*	0.816***	0.762***	0.716***	0.717***	0.241***	0.231***
	(0.042)	(0.042)	(0.034)	(0.082)	(0.035)	(0.033)
*Asset*	0.036	0.072	− 0.233***	0.051	− 0.066	0.780***
	(0.051)	(0.051)	(0.042)	(0.098)	(0.042)	(0.041)
*Leverage*	0.217**	0.393***	0.509***	− 0.030	0.457***	0.229***
	(0.091)	(0.092)	(0.074)	(0.190)	(0.077)	(0.073)
*Mtbt*	0.086**	0.029	0.178***	0.041	− 0.226***	− 0.183***
	(0.040)	(0.041)	(0.033)	(0.077)	(0.034)	(0.032)
*Lev*	0.017	0.251	− 0.621***	0.371	0.068	0.463**
	(0.249)	(0.252)	(0.203)	(0.485)	(0.209)	(0.200)
*ROA*	− 0.073	− 0.2269**	− 0.114	0.098	− 0.187**	− 0.047
	(0.094)	(0.094)	(0.075)	(0.193)	(0.078)	(0.075)
Year fixed effects	Yes	Yes	Yes	Yes	Yes	Yes
Constants	7.320***	6.533***	8.179***	5.232***	3.609***	5.611***
	(0.674)	(0.682)	(0.553)	(1.376)	(0.565)	(0.540)
*N*	1513	1640	1601	1232	1645	1645
*R*^*2*^	0.698	0.734	0.590	0.491	0.621	0.717
*F*	55.831	72.752	36.890	18.806	43.255	66.779

standard errors are presented in parentheses

^*^p < 0.1, **p < 0.05, ***p < 0.01

Secondly, we analyze the effects of carbon action–level information on corporate carbon emissions. *Oppo* has a positive effect on decreasing Scope 2 carbon emissions by 4.9%. *Managerexe* has a positive effect on decreasing Scope 1 carbon emissions by 5.1%. *Target* has a positive effect on decreasing Scope 3 carbon emissions by 7.1%. Compared to intensity targets, absolute targets decrease the aggregate effect of* Scope 1*, *Rin*, and *Ein* by 3.9%, 4.8%, and 4.3% respectively for a one-standard-deviation increase. In the emission reduction activities, *Incentiveemp* decreases the aggregate effect of* Total*,* Scope 1*, *Rin*, and *Ein* by 3.3%, 4.7%, 5.4%, and 5.5% respectively for a one-standard-deviation increase. Other emission reduction activities do less to reduce corporate carbon emissions. *Voluntary* has a positive effect on decreasing the aggregate effect of *Total* and* Scope 3* by 4.3% and 6.2% respectively for a one-standard-deviation increase. *Energy*, *Icp*, *Carbonmes*, *Thirty*, *Credit*, *Ets*, *Touch*, and *Value* have significant effects on corporate carbon emissions, but the role is the opposite.

Thirdly, we analyze the effects of financial-level information on corporate carbon emissions. *Currentratio* decreases the aggregate effect of* Scope 1*, *Rin*, and *Ein* by 9.5%, 10%, and 5% respectively for a one-standard-deviation increase. *Quickratio* decreases the aggregate effect of* Scope 3* by 11.7% for a one-standard-deviation increase. *Operprotio* decreases the aggregate effect of *Total*,* Scope 1*, *Scope 2*,* Scope 3*, *Rin*, and *Ein* by 20.6%, 22.7%, 31.5%, 16%, 9.4%, and 18.1% respectively for a one-standard-deviation increase. *Inventurn* decreases the aggregate effect of *Total*,* Scope 1*,* Scope 2*, *Rin*, and *Ein* by 4%, 3.2%, 8.3%, 4.3%, and 4.6% respectively for a one-standard-deviation increase. *Capitalstock* decreases the aggregate effect of *Total* and* Scope 3* by 13% and 19.6% respectively for a one-standard-deviation increase. *R&D* decreases the aggregate effect of* Scope 1*, *Rin*, and *Ein* by 7.1%, 9.8%, and 9.9% respectively for a one-standard-deviation increase. *Totassover* decreases the aggregate effect of *Rin* by 8.4% for a one-standard-deviation increase. *Mtbt* has an effect on relative carbon emissions, which decreases the aggregate effect of *Rin* and *Ein* by 17.6% and 13.2% respectively. *Asset and Lev* decrease the aggregate effect of* Scope 2* by 17.5% and 14% respectively. *ROA* decreases the aggregate effect of* Scope 1* and *Rin* by 6.1% and 7.3% respectively for a one-standard-deviation increase. *Operprotio* and *Capx* have a significant impact on carbon emissions across all ranges (Table 3).

### Sorting the importance of impact factors

**Fig. 3:**
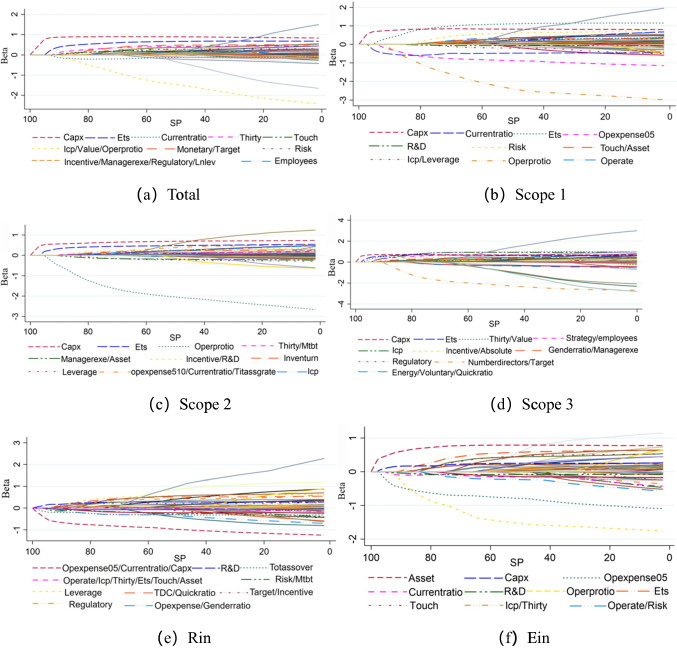
**a**–**f **The trend of coefficients with the decrease of SP

To identify the factors affecting carbon emissions, we selected the LASSO regression model and adopted the linear regression method of *L*_1_ regularization to make the eigenvalues of some influencing factors as 0 so as to achieve the purpose of sparsification and feature selection. SP is a sparse constraint that reflects the importance of each impact factor on carbon emissions. SP is in the range of 0–100 and gradually decreases from 100 to 0. At this time, the coefficients of impact factors also start to change from zero to non-zero. The variables with non-zero coefficients that enter the model first have the greatest impact on carbon emissions. We classified carbon emissions into absolute and relative quantities and tested the importance of impact factors from the absolute quantities of different ranges and the relative quantities of per revenues and per full-time equivalent employees. The results are shown in Fig. 3a–f. We list only the variables that entered the model the first ten times in the legend. To present a complete and more intuitive result, we give the LASSO path for all the variables that entered the model in Tables 5, 6, 7, 8, and 9. The results are presented in the Appendix.

Figure 2 shows that of all the impact factors, except the influence on *Ein*, *Capx* is the first variable to enter the model. Corporate capital expenditures are associated with more value-relevant activity and lead to more carbon emissions (Karim et al. 2021). Corporations with higher capital expenditure not only communicate more environmental impact information with the stakeholders, but also promote environmental activity to convey a positive image to their stakeholder (Zheng et al. 2020). For the Scope 1 carbon emissions, Scope 2 carbon emissions, and carbon emissions per revenues, *Currentratio* is an important influence factor. As corporate liquidity, the current ratio reflects the corporate short-term debt solvency. The greater the amount of corporate liquidity, the more it can assist in the reduction of carbon emissions (Chen et al. 2022). For the absolute carbon emissions, *Ets* is the second variable to enter the model. *Ets* is a cap-and-trade program that allows corporations regulated by the *Ets* to choose the most cost-effective way to manage their emissions through the purchase and sale of carbon allowances. Corporate adoption of this regulatory policy demonstrates its willingness to formulate strategies on carbon emissions that help corporations reduce emissions (Hossain and Farooque 2019). *Scope 1* and *Scope 2* are the corporate direct carbon emissions and indirect carbon emissions respectively associated with the purchase of electricity and energy, so *Opexpense* seems to be important and enters the model earlier. For the relative carbon emissions, Fig. 3e, f shows that *Opexpense* is critical. Energy consumption reflects the corporate operational efficiency. In the production of energy, corporations need to reduce carbon emissions significantly. Corporations may incur higher energy expenses by greater investments in environmental energy projects, which reflect lower emissions (Mahapatra et al. 2021). Furthermore, *Operate* is also an important impact factor. A firm’s boundary choice determines which emissions are under its control. We found that the operate boundary has a greater impact on carbon emissions than the finance boundary (Stanny 2018). Scope 3 emissions include indirect emissions that occur in the upstream and downstream of a company’s supply chain. Thus, in Fig. 3d, *Value* is the third variable to enter the model supply chain when corporations integrate climate-related issues into their business strategy. Carbon emissions in the supply chain are closely tied to business strategies, so *Strategy* is the fourth variable to enter the model. Only *R&D* does not enter the model of *Total* and *Scope 3*. Compared to other carbon emission ranges, *R&D* enters the model earlier. It can be seen that *R&D* is a key factor in corporate carbon emissions. *Thirty* tends to measure goods and services. Compared to the direct carbon emissions in *Scope 1*, the importance of *Scope 2* and *Scope 3* and relative quantities is higher. *Risk* is also included in the model, but *Oppo* does not enter any model. This is because companies focus more on risks than on opportunities when considering climate-related risks and opportunities (Gasbarro et al., 2017). *Icp* enters all the models. The implementation of the internal carbon price (ICP) contributes to enhancing the ability to implement and transform corporate environmental strategies and promotes the improvement of corporate carbon performance (Zhu et al. 2022). In Fig. 3a, b, c, f, *Touch* enters the model. We find that corporations’ participation in public policy is also an important factor influencing carbon emissions. When firms reach out to decision makers on taxation, regulation, and carbon regulation, they are the first to understand policy trends and engage in favorable emission reduction activities that cater to policies. *Incentive* can greatly affect corporate activities. In Fig. 3a, d, all incentive types enter the model. *Scope 2* and *Rin* are also influenced by *Incentive*. Furthermore, some carbon emission ranges are affected by a number of special factors. Profitability (*Operprotio*) has an impact on *Total*, *Scope 1*, *Scope 2*, and *Ein*. *Genderratio* and *Quickratio* have an impact only on *Scope 3* and *Rin*. *Monetary* has an impact only on *Total*.

### The regression of filtered impact factors to carbon emissions

Table 4 reports the estimated results of the fixed effects regression after optimal constraint intensity selection. For *Total*, *Dual*, *Opexpense*, *Opexpense05*, *TDC*, and *Numberdirectors* enter the model as firm-level impact factors. *Opexpense05* and *TDC* affect *Total* at a significant level of 1% and decrease the aggregate effect by 4% and 0.1% respectively. *Incentive*, *Employees*, *Managerexe*, *Monetary*, *Target*, *Intensity*, *Regulatory*, *Energy*, *Icp*, *Carbonmes*, *Thirty*, *Ets*, *Touch*, *Voluntary*, and *Value* enter the model as carbon action–level impact factors. *Employees*, *Target*, *Intensity*, *Regulatory*, *Energy*, *Icp*, *Thirty*, *Ets*, *Touch*, *Voluntary*, and *Value* affect *Total* at a significant level of 10%, but most of the carbon action–level factors play an opposite role in decreasing carbon emissions. *Currentratio*, *Operprotio*, *Inventurn*, *Capitalstock*, *Totassgrate*, *Capx*, *Asset*, *Leverage*, and *Lev* enter the model as financial-level impact factors. *Operprotio*, *Inventurn*, *Totassgrate*, *Asset*, and *Lev* affect *Total* at a significant level of 10%. *Operprotio* and *Inventurn* decrease the aggregate effect by 13.6% and 3.4% respectively.

**Table 4:** The regression of filtering the impact factors on carbon emissions

	(1)	(2)	(3)	(4)	(5)	(6)
	*Total*	*Scope 1*	*Scope 2*	*Scope 3*	*Rin*	*Ein*
Firm level						
*Strategy*				0.865***		
				(0.295)		
*Operate*		− 0.191**			− 0.456***	− 0.208***
		(0.081)			(0.113)	(0.065)
*Finance*			0.185***	− 0.151	− 0.339***	
			(0.071)	(0.175)	(0.125)	
*Team*		0.314***			0.154**	0.165***
		(0.072)			(0.063)	(0.059)
*CEO*		0.380***	− 0.057		0.148	0.183**
		(0.104)	(0.077)		(0.092)	(0.085)
*Founder*		− 0.555***	− 0.564***			
		(0.204)	(0.165)			
*Dual*	0.225***	0.421***		0.286**	0.153***	0.172***
	(0.074)	(0.0669)		(0.126)	(0.058)	(0.054)
*Boardamount*					− 0.505*	
					(0.295)	
*Opexpense*	0.376***	0.587***			0.778***	0.401***
	(0.141)	(0.120)			(0.123)	(0.098)
*Opexpense05*	− 0.339***	− 0.902***	− 0.130**		− 1.118***	− 0.797***
	(0.083)	(0.079)	(0.055)		(0.095)	(0.064)
*Opexpense510*					− 0.358***	
					(0.112)	
*Opexpense1015*					− 0.299**	
					(0.142)	
*TDC*	− 0.015***	− 0.020***			− 0.016***	− 0.018***
	(0.004)	(0.004)			(0.003)	(0.003)
*Nationalitymix*			0.385***	0.352		
			(0.136)	(0.306)		
*Genderratio*				− 1.889***	0.612*	0.783***
				(0.680)	(0.319)	(0.293)
*Numberdirectors*	0.015	0.04 3**		0.051	− 0.038**	
	(0.020)	(0.018)		(0.034)	(0.016)	
Carbon action level						
*Awareness*			0.180			
			(0.130)			
*Risk*		0.617***	− 0.100		0.356***	0.386***
		(0.088)	(0.113)		(0.077)	(0.072)
*Incentive*	− 0.015		0.326***	0.0558	0.058	0.098
	(0.147)		(0.083)	(0.243)	(0.089)	(0.081)
*Employees*	0.268***			0.349**	0.088	0.100
	(0.088)			(0.140)	(0.067)	(0.063)
*Managerexe*	0.159		0.103	0.037		
	(0.099)		(0.064)	(0.162)		
*Monetary*	0.080					
	(0.103)					
*Target*	0.396***			− 0.031	0.343***	0.064
	(0.122)			(0.261)	(0.125)	(0.088)
*Intensity*	0.150*	0.218***		0.660***	0.051	0.229***
	(0.085)	(0.069)		(0.171)	(0.087)	(0.064)
*Absolute*				0.399**	− 0.204**	
				(0.170)	(0.085)	
*Regulatory*	0.169**	0.170**		0.486***	0.088	0.091
	(0.077)	(0.070)		(0.124)	(0.062)	(0.057)
*Incentiveemp*		− 0.313***	0.084		− 0.175***	− 0.204***
		(0.072)	(0.056)		(0.064)	(0.059)
*Energy*	0.242***			0.182	0.120*	
	(0.079)			(0.127)	(0.063)	
*Icp*	0.509***	0.738***	− 0.148	0.405**	0.831***	0.744***
	(0.129)	(0.117)	(0.092)	(0.205)	(0.103)	(0.096)
*Carbonmes*	0.098	0.108	0.302**		0.103	0.089
	(0.195)	(0.180)	(0.139)		(0.162)	(0.148)
*Thirty*	0.480***	0.093	0.217***	0.852***	0.241***	0.174***
	(0.082)	(0.075)	(0.058)	(0.133)	(0.065)	(0.061)
*Credit*				0.173		
				(0.157)		
*Ets*	0.622***	1.146***	0.476***	0.673***	− 0.009	0.622***
	(0.084)	(0.077)	(0.060)	(0.140)	(0.014)	(0.084)
*Touch*	0.239**	0.247***		0.135	0.263***	0.271***
	(0.093)	(0.086)		(0.157)	(0.075)	(0.070)
*Voluntary*	− 0.223***			− 0.400***		
				(0.052)		
*Value*	0.396***			0.792***		
	(0.112)			(0.222)		
Financial level						
*Currentratio*	− 0.103	− 0.464***	0.462***		− 0.277***	− 0.067
	(0.084)	(0.086)	(0.058)		(0.074)	(0.069)
*Quickratio*		0.529***		− 0.399***	0.695***	0.615***
		(0.103)		(0.152)	(0.097)	(0.081)
*Netprosales*		0.495*	0.040		2.050***	
		(0.279)	(0.216)		(0.438)	
*Operprotio*	− 1.622***	− 2.900***	− 2.307***		− 0.955***	− 1.668***
	(0.228)	(0.247)	(0.187)		(0.228)	(0.198)
*Receiturntio*			1.368**		− 0.670	
			(0.621)		(0.659)	
*Inventurn*	− 0.161***	− 0.191***	− 0.262***		− 0.192***	− 0.220***
	(0.056)	(0.054)	(0.048)		(0.053)	(0.051)
*Capitalstock*	0.168				0.988*	
	(0.119)				(0.58)	
*Totassgrate*	0.061***		0.004		0.073***	0.045**
	(0.023)		(0.003)		(0.020)	(0.018)
*R&D*		− 0.072***	0.067***	0.100***	− 0.068***	− 0.082***
		(0.013)	(0.011)	(0.019)	(0.012)	(0.011)
*Totassover*					− 0.178*	
					(0.093)	
*Capx*	0.889***	0.796***	0.741***	0.765***	0.290***	0.273***
	(0.033)	(0.031)	(0.024)	(0.049)	(0.032)	(0.027)
*Asset*	0.082**	0.180***	− 0.266***		− 0.050	0.794***
	(0.039)	(0.035)	(0.028)		(0.038)	(0.029)
*Leverage*	0.127	0.369***	0.260***		0.481***	0.168***
	(0.083)	(0.077)	(0.039)		(0.049)	(0.062)
*Mtbt*			0.106***		− 0.254***	− 0.215***
			(0.023)		(0.030)	(0.025)
*Lev*	0.366*	0.291		0.821***		0.585***
	(0.192)	(0.182)		(0.203)		(0.163)
*ROA*					− 0.161**	
					(0.074)	
Constants	7.425***	7.164***	9.237***	5.651***	4.178***	5.284***
	(0.402)	(0.369)	(0.270)	(0.828)	(0.500)	(0.382)
Year fixed effects	Yes	Yes	Yes	Yes	Yes	Yes
*N*	1700	2209	2180	1677	2004	2144
*R*^2^	0.669	0.7173	0.591	0.454	0.606	0.705
*F*	93.265	148.892	91.170	42.728	58.808	125.510

standard errors are presented in parentheses

^*^p < 0.1, **p < 0.05, ***p < 0.01

For *Scope 1*, *Operate*, *Team*, *CEO*, *Founder*, *Dual*, *Opexpense*, *Opexpense05*, *TDC*, and *Numberdirectors* enter the model as firm-level impact factors. *Operate*, *Founder*, *Opexpense05*, and *TDC* affect *Scope 1* at a significant level of 1% and decrease the aggregate effect by 3.1%, 3.3%, 10.6%, and 0.1% respectively. *Risk*, *Intensity*, *Regulatory*, *Incentiveemp*, *Icp*, *Carbonmes*, *Thirty*, *Ets*, and *Touch* enter the model as carbon action–level impact factors. *Risk*, *Intensity*, *Regulatory*, *Incentiveemp*, *Icp*, *Ets*, and *Touch* affect *Scope 1* at a significant level of 1%, but most of the carbon action–level factors have an opposite role in decreasing carbon emissions. *Currentratio*, *Quickratio*, *Netprosales*, *Operprotio*, *Inventurn*, *R&D*, *Capx*, *Asset*, *Leverage*, and *Lev* enter the model as financial-level impact factors. *Currentratio*, *Quickratio*, *Netprosales*, *Operprotio*, *Inventurn*, *R&D*, *Capx*, *Asset*, and *Leverage* affect *Scope 1* at a significant level of 10%. *Currentratio*, *Operprotio*, *Inventurn*, and *R&D* decrease the aggregate effect by 9.1%, 21.5%, 3.5%, and 7.4% respectively.

For *Scope 2*, *Finance*, *CEO*, *Founder*, *Opexpense05*, and *Nationalitymix* enter the model as firm-level impact factors. *Finance*, *Founder*, *Opexpense05*, and *Nationalitymix* affect *Scope 2* at a significant level of 5%. *Founder* and *Opexpense05* decrease the aggregate effect by 4.6% and 2.1% respectively. *Awareness*, *Risk*, *Incentive*, *Managerexe*, *Incentiveemp*, *Icp*, *Carbonmes*, *Thirty*, and *Ets* enter the model as carbon action–level impact factors. *Incentive*, *Carbonmes*, *Thirty*, and *Ets* affect *Scope 2* at a significant level of 5%, but these factors have an opposite role in decreasing carbon emissions. *Currentratio*, *Netprosales*, *Operprotio*, *Receiturntio*, *Inventurn*, *Totassgrate*, *R&D*, *Capx*, *Asset*, *Leverage*, and *Mtbt* enter the model as financial-level impact factors. *Currentratio*, *Operprotio*, *Receiturntio*, *Inventurn*, *R&D*, *Capx*, *Asset*, *Leverage*, and *Mtbt* affect *Scope 2* at a significant level of 5%. *Operprotio*, *Inventurn*, and *Asset* decrease the aggregate effect by 26.7%, 7.6%, and 20% respectively.

For *Scope 3*, *Strategy*, *Finance*, *Dual*, *Nationalitymix*, *Genderratio*, and *Numberdirectors* enter the model as firm-level impact factors. *Strategy*, *Dual*, and *Genderratio* affect *Scope 3* at a significant level of 5%. *Genderratio* decreases the aggregate effect by 128.3%. *Incentive*, *Employees*, *Managerexe*, *Target*, *Intensity*, *Absolute*, *Regulatory*, *Energy*, *Icp*, *Thirty*, *Credit*, *Ets*, *Touch*, *Voluntary*, and *Value* enter the model as carbon action–level impact factors. *Employees*, *Intensity*, *Absolute*, *Regulatory*, *Icp*, *Thirty*, *Ets*, *Voluntary*, and *Value* affect *Scope 3* at a significant level of 5%. Only *Voluntary* decreases the aggregate effect by 6.1%. *Quickratio*, *R&D*, *Capx*, and *Lev* enter the model as financial-level impact factors and affect *Scope 3* at a significant level of 1%. Only *Quickratio* decreases the aggregate effect by 7.2%.

For *Rin*, *Operate*, *Finance*, *Team*, *CEO*, *Dual*, *Boardamount*, *Opexpense*, *Opexpense05*, *Opexpense510*, *Opexpense1015*, *TDC*, *Genderratio*, and *Numberdirectors* enter the model as firm-level impact factors. Except for *CEO*, all the factors affect *Rin* at a significant level of 10%. *Operate*, *Finance*, *Boardamount*, *Opexpense05*, *Opexpense510*, *Opexpense1015*, *TDC*, and *Numberdirectors* decrease the aggregate effect by 9.6%, 5.9%, 3%, 16.8%, 3.5%, 130.7%, 0.1%, and 0.7% respectively. *Risk*, *Incentive*, *Employees*, *Target*, *Intensity*, *Absolute*, *Regulatory*, *Incentiveemp*, *Energy*, *Icp*, *Carbonmes*, *Thirty*, *Ets*, and *Touch* enter the model as carbon action–level impact factors. *Risk*, *Target*, *Absolute*, *Incentiveemp*, *Energy*, *Icp*, *Thirty*, and *Touch* affect *Rin* at a significant level of 10%. *Absolute* and *Incentiveemp* decrease the aggregate effect by 4.9% and 4.2% respectively. *Currentratio*, *Quickratio*, *Netprosales*, *Operprotio*, *Receiturntio*, *Inventurn*, *Capitalstock*, *Totassgrate*, *R&D*, *Totassover*, *Capx*, *Asset*, *Leverage*, *Mtbt*, and *ROA* enter the model as financial-level impact factors. Except for *Receiturntio* and *Asset*, all the factors affect *Rin* at a significant level of 10%. *Currentratio*, *Operprotio*, *Inventurn*, *R&D*, *Totassover*, *Mtbt*, and *ROA* decrease the aggregate effect by 7.9%, 10.3%, 5.2%, 10.1%, 6.7%, 19.7%, and 6.3% respectively.

For *Ein*, *Operate*, *Team*, *CEO*, *Dual*, *Opexpense*, *Opexpense05*, *TDC*, and *Genderratio* enter the model as firm-level impact factors and all affect *Rin* at a significant level of 5%. *Operate*, *Opexpense05*, *Opexpense510*, and *TDC* decrease the aggregate effect by 4%, 11%, and 0.2% respectively. *Risk*, *Incentive*, *Employees*, *Target*, *Intensity*, *Regulatory*, *Incentiveemp*, *Icp*, *Carbonmes*, *Thirty*, *Ets*, and *Touch* enter the model as carbon action–level impact factors. *Risk*, *Intensity*, *Incentiveemp*, *Icp*, *Thirty*, and *Touch* affect *Ein* at a significant level of 1%. Only *Incentiveemp* decreases the aggregate effect by 4.5%. *Currentratio*, *Quickratio*, *Operprotio*, *Inventurn*, *Totassgrate*, *R&D*, *Capx*, *Asset*, *Leverage*, *Mtbt*, and *Lev* enter the model as financial-level impact factors. Except for *Currentratio*, all the factors affect *Rin* at a significant level of 5%. *Operprotio*, *Inventurn*, *R&D*, and *Mtbt* decrease the aggregate effect by 16.5%, 5.4%, 11.2%, and 15.3% respectively.

### Partial samples analysis

#### Subsample analysis for the carbon-intensive sector

Table 5 reports the estimated results of the regression of impact factors in different sectors. The impact factors affecting different sectors vary widely. Corporations from the carbon-intensive sector are subject to higher climate change–related risks, and therefore, we may expect these corporations to provide more information about climate change–related strategies than corporations from the low-carbon sector. Inspired by Zhou et al. (2018), we define chemicals, gas and electrical utilities, oil and gas, coal mining, pipelines, steel, and transportation as belonging to the carbon-intensive sector. Others belong to the non-carbon-intensive sector.

**Table 5:** The regression of impact factors in different sectors

	*Total*		
	Carbon-intensive sector		Non-carbon-intensive sector
*Capx*	0.909***	*Capx*	0.928***
	(0.026)		(0.031)
*Currentratio*	0.042	*Ets*	0.654***
	(0.072)		(0.084)
*Operprotio*	− 1.674***	*Value*	0.368***
	(0.166)		(0.112)
*Ets*	0.619***	*Monetary*	0.104
	(0.070)		(0.103)
*Numberdirectors*	0.035**	*Incentive*	− 0.100
	(0.017)		(0.146)
*Asset*	0.077**	*Managerexe*	0.111
	(0.031)		(0.099)
*Icp*	0.735***	*Thirty*	0.478***
	(0.110)		(0.093)
*Touch*	0.392***	*Touch*	0.280***
	(0.080		(0.093)
*Thirty*	0.385***	*Currentratio*	− 0.157*
	(0.067)		(0.084)
*Strategy*	0.417***	*Risk*	0.552***
	(0.122)		(0.101)
*Carbonmes*	0.083	*Target*	0.333**
	(0.162)		(0.120)
*Totassgrate*	− 0.004	*Regulatory*	0.172^**^
	(0.003)		(0.076)
*Dual*	0.244***	*Employees*	0.288^***^
	(0.063)		(0.087)
*Energy*	0.126*	*Operprotio*	− 1.539***
	(0.066)		(0.217)
*Target*	0.659***	*Lev*	0.354*
	(0.096)		(0.188)
*Lev*	0.733***	*Leverage*	0.132*
	(0.107)		(0.082)
*Genderratio*	− 0.114	*Icp*	0.599***
	(0.334)		(0.128)
*Opexpense05*	− 0.287***	*Nationalitymix*	0.227
	(0.064)		(0.188)
*Voluntary*	− 0.167**	*Intensity*	0.141*
	(0.068)		(0.085)
*Capitalstock*	0.164	*Energy*	0.222***
	(0.121)		(0.077)
*Intensity*	0.158**	*Inventurn*	− 0.162***
			(0.056)
	(0.072)	*TDC*	− 0.016***
			(0.004)
Constants	7.304***	Constants	7.585***
	(0.435)		(0.270)
Year fixed effects	Yes	Year fixed effects	Yes
*N*	2505	*N*	1699
*R*^2^	0.662	*R*^2^	0.665
*F*	161.841	*F*	114.149

standard errors are presented in parentheses

^*^p < 0.1, **p < 0.05, ***p < 0.01

For the carbon-intensive sector, financial-level impact factors are of high importance. *Capx* is the impact factor with the highest importance. Higher capital expenditures are not conducive to reducing carbon emissions. Karim et al. (2021) show that capital expenditure leads to more carbon emissions. It is punishable by the market because non-green investments in capital expenditures may increase carbon emissions. Green investments may not offer any benefits in the short term, leading to a more negative market reaction (Lee et al. 2015). This is followed by operation capability. The coefficient of *Operprotio* is − 1.674 and is significant at the level of 1%. *Operprotio* decreases the aggregate effect of *Total* by 14%. *Ets* is the third important factor. It is easier for corporations to carbon trade after participating in the ETS. A variety of corporate carbon actions also have a significant impact on corporate carbon emissions, including *Icp*, *Touch*, *Thirty*, *Energy*, *Target*, and *Intensity*. *Voluntary* can reflect that corporations with voluntary emission reduction awareness are more conducive to carbon emission reduction, and it decreases the aggregate effect of *Total* by 3%. In the impact factors of firm level, although *Numberdirector*, *Strategy*, and *Dual* affect corporate carbon emissions, they cannot promote a reduction in the number of carbon emissions. The coefficient of *Opexpense05* is − 0.287 and is significant at the level of 1%. *Opexpense05* decreases the aggregate effect of *Total* by 3.4%.

For the non-carbon-intensive sector, *Capx* first enters the model. The greatest impact is also by financial-level factors. Corporate carbon actions have a significant impact on corporate carbon emissions. *Ets*, *Value*, *Thirty*, *Touch*, *Risk*, *Target*, *Regulatory*, *Employees*, *Icp*, *Intensity*, and *Energy* all have a significant influence on carbon emissions. In addition, the impact factors of financial level, debt-paying ability, operation capability, and profitability deserve attention from corporations. *Currentratio*, *Operprotio*, and *Inventurn* can decrease the aggregate effect of *Total* by 3.5%, 12.9%, and 3.4% respectively. In the impact factors of firm level, the coefficient of *TDC* is − 0.016 and is significant at the level of 1%. *TDC* decreases the aggregate effect of *Total* by 0.1%.

#### Subsample analysis for region

According to the geographic distribution of the United States Census Bureau, we divided the 50 US states into the Northeast region, South region, Mid-west region, and West region. Then, we explored the factors affecting corporate carbon emissions in these four regions. The results are reported in Table 6. For Northeast region corporations, only four variables enter the model. *Monetary*, *Ets*, and *Capx* have significant influences on corporate carbon emissions. For Mid-west region corporations, the impact factors are focused mainly on carbon actions. The factor of *Dual* at the firm level and the factor of *Operprotio* and *Capx* at the financial level have a significant impact on corporate carbon emissions. For South region corporations, there are many impact factors. The factor of *Opexpense05* and of *TDC* at firm level decrease the aggregate effect of *Total* by 5.2% and 0.1%. The factors of *Awareness*, *Employees*, *Target*, *Regulatory*, *Icp*, *Thirty*, *Ets*, *Touch*, *Voluntary*, and *Value* at the carbon action level have a significant impact on corporate carbon emissions. *Voluntary* decreases the aggregate effect of *Total* by 3.4%. The factors of *Capx*, *Mtbt*, and *Lev* at the financial level have a significant impact on corporate carbon emissions. *Mtbt* decreases the aggregate effect of *Total* by 3.9%. For West region corporations, the factor of *Strategy* at the firm level has a significant impact on corporate carbon emissions. The factors of *Risk*, *Employees*, *Monetary*, *Thirty*, and *Ets* at the carbon action level have a significant impact on corporate carbon emissions. The factors of *Currentratio*, *Capx*, and *Asset* at the financial level have a significant impact on corporate carbon emissions. *Currentratio* decreases the aggregate effect of *Total* by 8.1%.

**Table 6:** The regression of impact factors in regions

	*Total*			
	Northeast	Mid-west	South	West
*Strategy*				0.690***
				(0.123)
*Dual*		0.203***		
		(0.070)		
*Opexpense*			0.435***	
			(0.137)	
*Opexpense05*			− 0.445***	
			(0.082)	
*TDC*			− 0.018***	
			(0.004)	
*Nationalitymix*	− 0.171			
	(0.172)			
*Genderratio*			− 0.335	
			(0.385)	
*Awareness*			0.536***	
			(0.110)	
*Risk*		0.652***		0.603***
		(0.088)		(0.083)
*Incentive*			0.052	
			(0.131)	
*Employees*		0.148**	0.277***	0.157**
		(0.071)	(0.082)	(0.070)
*Monetary*	0.431***		0.153	0.300***
	(0.072)		(0.096)	(0.071)
*Target*		0.572***	0.436***	
		(0.091)	(0.103)	
*Regulatory*		0.287***	0.254***	
		(0.07)	(0.075)	
*Icp*		0.768***	0.633***	
		(0.121)	(0.126)	
*Carbonmes*			0.253	
			(0.191)	
*Thirty*		0.397***	0.425***	0.590***
		(0.075)	(0.079)	(0.0700)
*Credit*			− 0.097	
			(0.094)	
*Ets*	0.822***	0.677***	0.693***	0.786***
	(0.077)	(0.078)	(0.082)	(0.075)
*Touch*		0.433***	0.199**	
		(0.085)	(0.091)	
*Voluntary*			− 0.189**	
			(0.079)	
*Value*		0.430***	0.420***	
		(0.103)	(0.108)	
*Currentratio*			− 0.099	− 0.365***
			(0.081)	(0.063)
*Operprotio*		− 1.617***		
		(0.152)		
*Receiturntio*			0.178	
			(0.844)	
*Totassgrate*				− 0.003
				(0.004)
*Capx*	1.046***	0.843***	0.978***	0.851***
	(0.023)	(0.022)	(0.033)	(0.025)
*Asset*			− 0.031	0.061**
			(0.035)	(0.030)
*Mtbt*			− 0.065**	
			(0.028)	
*Lev*			0.648***	
			(0.134)	
*Constant*	4.718***	7.086***	7.236***	4.587***
	(0.221)	(0.191)	(0.485)	(0.263)
*N*	2731	2391	1811	2920
*R*^*2*^	0.562	0.624	0.677	0.576
*F*	248.947	207.290	120.355	197.161

standard errors are presented in parentheses

^*^p < 0.1, **p < 0.05, ***p < 0.01

## Discussion and policy implications

### Discussion and conclusion

Corporations are gradually becoming major actors in the fight against climate change. Governments, investors, and stakeholders are also beginning to value the environmental responsibilities of corporations. Therefore, it is necessary to identify the key drivers affecting corporate carbon reduction to implement effective emission reduction measures. The existing literature mostly involves research into the drivers of carbon emission reduction based on specific assumptions (Mahapatra et al. 2021). This method can only explore the degree of importance of the drivers and cannot distinguish the relative importance of the drivers. The LASSO regression differs from the general regression model in that not only can it empirically test the impact of firm-level, carbon action–level, and financial-level information on corporate carbon emissions, but it also ranks their importance and thus identifies the most influential driving factors of corporate carbon emissions.

This paper used CDP database questionnaire data, BoardEx data, and Compustat data to select a sample of 4016 US-listed corporations from 2009–2019. The LASSO regression model was then used to prioritize the most important factors affecting absolute carbon intensity (*Total*, *Scope 1*, *Scope 2*, and *Scope 3*) and relative carbon intensity (*Rin* and *Ein*). There is a further significant analysis of the important factors which are screened out. The results show that *Capx* is the most important factor affecting corporate carbon emissions. Although *Capx* does not enter the model first for *Ein*, it is the second variable to enter the model, which indicates that the impact is also very high. However, *Capx* has a negative impact on decreasing corporate carbon emissions. The result is consistent with Karim et al. (2021), who find that capital expenditure leads to more carbon emissions. Capital expenditures reflect more value-relevant activity, which causes an increase in corporate carbon emissions. While corporations with higher carbon emissions will provide more information on carbon emissions to reduce negative market reactions, these corporations are still punished by the market. Even green capital expenditures do not pay off in the market (Lee et al. 2015). Thus, we argue that corporations should reduce carbon emissions by reasonably reducing capital expenditures. For absolute carbon emissions, *Ets* is the most important impact factor, but we find that *ETS* is not conducive to reducing carbon emissions. Unlike the EU ETS, which is a mandatory program, the USA is a contracted-based and voluntary market for trading carbon allowances. It is a dynamic market, and the determinant of carbon allowance trading is energy prices, particularly influenced by the price of coal (Kim and Koo 2010). The USA implements many other strategies for reducing carbon emissions (Villoria-Sáez et al. 2016); only the ETS does not play a great role. *Scope 1* and *Scope* 2 are direct and indirect emissions associated with corporations, so financial-level factors play a greater role. For *Scope 1*, corporate debt-paying ability is more important. Highly indebted corporations struggle with onerous debt responsibilities, which limit the implementation of management strategies to reduce carbon emissions (Sun et al. 2022). Thus, we argue that the higher corporate debt-paying ability, the more focus is on carbon emission reduction activities. For *Scope 2*, corporate operation capability is more important. *Operprotio* is the third factor that enters the model and has a negative relationship with carbon emissions. This finding is consistent with Ganda and Milondzo (2018). Furthermore, research and development (R&D) is also a factor worth paying attention to. Corporations with R&D are more likely to be associated with improved environmental performance (Li et al. 2021). Many studies find that R&D contributes to a reduction of carbon emissions (Li et al. 2021; Petrović and Lobanov 2020; Koçak and Ulucak 2019). We also have consistent results that R&D can reduce both Scope 1 emissions and relative carbon emissions. However, we find R&D cannot reduce Scope 2 and Scope 3 emissions. Scope 2 emissions are the indirect emissions from electricity, which are sources from corporate purchases and consumption. Scope 3 emissions are not corporate owned or controlled. Thus, R&D activities that are applied to reduce Scope 1 emissions can contribute to reducing emissions. For *Scope 3*, corporate internal incentive policies and emission reduction behaviors are important. *Thirty*, *Value*, *Strategy*, *Employees*, *Icp*, and *Incentive* are all among the top ten variables that enter the model. For relative carbon emissions, the financial-level factors’ debt-paying ability can be used as a reference indicator for the impact of corporate carbon emissions. Energy consumption intensity also enters the model earlier. Especially, when the energy percentage of the total operational spend is more than 0% but less than or equal to 5%, corporate carbon emissions can be most affected. Mahapatra et al. (2021) have also studied the impact of energy consumption intensity on corporate carbon emissions. Our finding is consistent that corporations concerned about carbon emissions have lower energy consumption intensity. In the analysis of the impact on *Total*, *Scope 1*, *Rin*, and *Ein*, *Risk* enters the model. It can be seen that carbon-risk awareness has a greater impact on the corporate total emission amount and the relative emission amount. Further, in the firms with carbon-intensive sector and non-carbon-intensive sector, the most important factor is still *Capx. Ets* is the second factor entering the model. However, firms with carbon-intensive sector are mainly influenced by the factors of financial level. Firms with non-carbon-intensive sector are mainly influenced by the factors of carbon action level. In the partial sample analysis of the region, we find that for corporations in the Northeast and Mid-west, the factors of carbon action level have a greater impact. For corporations in the South, impact factors are the most. Firm level, carbon action level, and financial level all have an influence. For corporations in the West, the impact factors are mainly focused on carbon action level and financial level. Only *Strategy* plays an influential role at the firm level.

### Policy implications

Based on our study, we offer important suggestions for managers and policymakers. First of all, *Capx* has a positive effect on the absolute carbon emissions of *Total*, Scope 1, Scope 2, and Scope 3 emissions. Thus, it is essential to appropriately reduce corporate capital expenditure. Corporate capital expenditure increases the carbon footprint of activities related to increased value. Corporations with greater capital expenditure can improve the transparency of their corporate carbon information by providing more carbon disclosures; more carbon emission information minimizes market penalties for corporate emissions (Matsumura et al. 2014). For policymakers, it means that they can increase the level of corporate carbon disclosure in the annual reports. Meanwhile, managers need to enhance internal governance to ensure that any carbon emissions caused by capital expenditure are fully communicated to the stakeholders and to improve the relationship between capital expenditure and carbon emission disclosures to promote lower corporate carbon emissions.

Secondly, for Scope 1 and Scope 2 emissions, the factors of *Currentratio* and *R&D* have important effects. Managers should pay more attention to corporate debt-paying ability to ensure a reduction in corporate carbon emissions. Tackling climate change challenges will impose additional costs and constraints on corporations. Thus, to ensure competitiveness, corporations should have an innovative ability. R&D is unlikely to make a decent profit in the short term, but investing in green technology, R&D can reduce carbon emissions and lead to positive financial outcomes (Lee et al. 2015). Regarding policymakers, they can not only develop fiscal incentives to encourage corporate R&D but can also cooperate with corporations to alleviate corporate pressure. Managers should comprehensively measure corporate environmental responsibility and financial performance to avoid missing business and profit opportunities as a result of insufficient information. They should also focus on investing in environmental technologies and green R&D.

Thirdly, for Scope 3 emissions, *ETS*, *Thirty*, *Value*, and *Employees* all have a significant impact on Scope 3 emissions. However, although Scope 3 emissions are affected by more corporate carbon actions, these impacts have not contributed to the reduction of Scope 3 emissions. Efforts to reduce carbon emissions are always incompatible with substantial environmental responsibility and economic outcomes. Chowdhury et al. (2018) argue that passive or symbolic carbon reduction actions are not effective and cannot reduce carbon emissions. For policymakers, they can strengthen the substantive role of emission reduction actions by changing the direction of policy supervision and distributing policy benefits. For managers, they should reduce these symbolic emission reduction actions and strive to integrate emission reduction actions into their business strategies and achieve them.

Fourthly, for the relative carbon emissions of *Rin* and *Ein*, energy consumption intensity is another impact factor we are concerned with. We compare the different proportions of energy consumption intensity and find that only when the energy percentage of the total operational spend is less than or equal to 10% does it have an impact on corporate carbon emissions. *Opexpense05* and *Opexpense510* all enter the model, but *Opexpense1015* is excluded. *Opexpense* only enters one model of *Rin*, which indicates that having a higher proportion of energy consumption is not always better. For policymakers, they can require corporations to disclose proportions of energy consumption intensity in annual reports to play a monitoring role, while managers can try to maintain the corporate energy percentage of the total operational spend of more than 5% but less than or equal to 10% to promote lower carbon emissions.

For corporations, carbon emission reduction as a corporate strategy is affected by a combination of factors. Although the LASSO regression used in this paper explores the factors affecting corporate carbon emissions in multiple dimensions, it is still limited by the linear regression model. Therefore, future research can consider incorporating nonlinear analysis techniques into studies to complement existing studies. Secondly, this study is limited by micro-data and does not include macro-level data, such as the economic development capacity of each region, which we argue is also an effective impact factor affecting corporate carbon emissions.

## Data Availability

Data is not available at current stage.

## Appendix 1

See Tables 7, 8, 9, 10, 11, and 12.

**Table 7:** LASSO pathways for *Total*

Knot	ID	$\lambda$	*s*	*L*_1_ norm	EBIC	*R*^2^	Action
1	1	5517.94631	1	0.00000	2755.76892	0.0000	Added cons
2	2	5027.74717	2	0.10806	2617.97766	0.0918	Added *Capx*
3	12	1983.04708	3	0.79144	1805.64791	0.4719	Added *Ets*
4	15	1500.10240	4	1.13789	1677.72657	0.5173	Added *Currentratio*
5	16	1366.83743	5	1.31971	1636.03526	0.5328	Added *Thirty*
6	17	1245.41136	6	1.51060	1597.18374	0.5471	Added *Touch*
7	18	1134.77245	9	1.75999	1575.08590	0.5606	Added *Icp*, *Value*, *Operprotio*
8	20	942.10802	11	2.45516	1495.31755	0.5875	Added *Monetary*,* Target*
9	21	858.41374	12	2.79084	1461.48054	0.5988	Added *Risk*
10	22	782.15463	16	3.14125	1452.36682	0.6094	Added *Incentive*, *Managerexe*, *Regulatory*,* Lev*
11	23	712.67017	16	3.48676	1414.39851	0.6169	Added *Employees*,* Energy*
12	27	491.21590	18	4.58965	1327.77868	0.6441	Added *Numberdirectors*
13	28	447.57764	19	4.81331	1316.93000	0.6485	Added* Leverage*
14	29	407.81607	21	5.05315	1313.36793	0.6530	Added *Dual, Opexpense05*
15	30	371.58682	24	5.27633	1317.18817	0.6575	Added *TDC*, *Intensity*, *Totassgrate*
16	31	338.57607	25	5.49437	1306.15737	0.6618	Added *Asset*
17	32	308.49790	29	5.72336	1320.65486	0.6656	Added *Opexpense*, *Carbonmes*, *Voluntary*, *Inventurn*
18	37	193.74567	30	6.97881	1263.84806	0.6796	Added *Capitalstock*
19	38	176.53384	32	7.27200	1271.71553	0.6813	Added *CEO*, *Nationalitymix*
20	39	160.85106	34	7.60613	1280.09707	0.6829	Added *Absolute*, *Netprosales*
21	41	133.54138	35	8.37667	1274.02971	0.6858	Added *Finance*
22	42	121.67793	36	8.74247	1276.34024	0.6870	Added *Genderratio*
23	43	110.86840	38	9.14167	1286.41778	0.6882	Added *Incentiveemp*, *Mtbt*
24	46	83.86788	39	10.19685	1280.98982	0.6909	Added *Oppo*
25	48	69.62859	40	10.79931	1282.54349	0.6922	Added *R&D*
26	49	63.44297	41	11.07632	1287.83964	0.6928	Added *Quickratio*
27	51	52.67147	43	11.57120	1299.58989	0.6936	Added *Founder*, *Totassover*
28	52	47.99228	44	11.80641	1305.85798	0.6939	Added *Team*
29	54	39.84404	45	12.22483	1311.17737	0.6945	Added *ROA*
30	57	30.14055	47	12.80521	1324.28519	0.6950	Added *Opexpense510*, *Receiturntio*
31	58	27.46295	49	13.00814	1339.39044	0.6952	Added *Operate*,* Opexpense1015*
32	62	18.92915	50	13.70232	1345.12299	0.6956	Added *Boardamount*
33	67	11.88805	51	14.29956	1351.80879	0.6960	Added *Incentive*
34	72	7.46604	53	14.67710	1367.19187	0.6960	Added *Strategy*, *Awareness*
35	73	6.80278	54	14.74010	1375.06975	0.6960	Added *Credit*

**Table 8:** LASSO pathways for *Scope 1*

Knot	ID	$\lambda$	*s*	*L*_1_ norm	EBIC	*R*^2^	Action
1	1	6226.06637	1	0.00000	3392.02790	0.0000	Added cons
2	2	5672.95980	2	0.11340	3267.31790	0.0777	Added *Capx*
3	8	3246.27617	3	0.66750	2728.62362	0.3391	Added *Currentratio*
4	11	2455.68887	4	1.17844	2518.16657	0.4215	Added *Ets*
5	12	2237.53224	5	1.47766	2436.40007	0.4523	Added *Opexpense05*
6	13	2038.75604	6	1.81295	2360.08408	0.4797	Added *Operprotio*
7	16	1542.24418	7	2.96207	2146.49237	0.5455	Added *R&D*
8	18	1280.39821	8	3.51338	2042.84332	0.5754	Added *Risk*
9	19	1166.65116	10	3.84275	2001.94497	0.5898	Added *Touch*,* Asset*
10	20	1063.00909	12	4.19567	1961.79060	0.6036	Added *Icp*,* Leverage*
11	21	968.57429	13	4.57427	1915.15210	0.6166	Added *Operate*
12	23	804.12739	16	5.25526	1847.61136	0.6374	Added *Dual*, *TDC*,* Thirty*
13	24	732.69093	17	5.57410	1809.41360	0.6474	Added *Regulatory*
14	26	608.29288	18	6.15321	1742.04717	0.6632	Added *Team*
15	27	554.25382	20	6.51104	1721.55934	0.6706	Added *Intensity*, *Quickratio*
16	28	505.01544	21	6.86971	1696.48804	0.6772	Added *Numberdirectors*
17	29	460.15126	22	7.21933	1675.50343	0.6828	Added *Carbonmes*
18	30	419.27269	24	7.57281	1665.82069	0.6877	Added *CEO*,* Lev*
19	31	382.02566	25	7.94276	1651.11495	0.6920	Added *Sales*
20	33	317.16441	26	8.79891	1620.98950	0.6991	Added *Opexpense*
21	34	288.98840	28	9.26739	1619.02789	0.7023	Added *Founder*, *Incentiveemp*
22	35	263.31547	29	9.71586	1611.25707	0.7052	Added *Inventurn*
23	39	181.49314	32	11.30241	1589.19758	0.7133	Added *Genderratio*, *Managerexe*
24	41	150.67879	33	12.05485	1580.63523	0.7162	Added *Incentive*
25	42	137.29290	36	12.44970	1596.42897	0.7176	Added *Opexpense510*, *Target*,* Credit*
26	44	113.98298	37	13.18276	1591.04855	0.7199	Added Finance
27	46	94.63067	38	13.91461	1587.96617	0.7271	Added *Receiturntio*
28	48	78.56405	40	14.61440	1595.93925	0.7231	Added *Nationalitymix*,* ROA*
29	49	71.58463	41	14.96853	1600.52083	0.7236	Added *Totassgrate*
30	51	59.43082	42	15.59435	1603.23417	0.7245	Added *Opexpense1015*
31	53	49.34051	47	16.22268	1638.35489	0.7253	Added *Oppo*, *Monetary*, *Value*, *Capitalstock*, *Totassover*
32	54	44.95724	48	16.56809	1644.06074	0.7257	Added *Voluntary*
33	56	37.32429	49	17.18601	1648.54996	0.7262	Added *Boardamount*
34	57	34.00850	50	17.47806	1655.08536	0.7265	Added *Mtbt*
35	60	25.72618	52	18.23608	1667.89114	0.7270	Added *Awareness*,* Employees*
36	63	19.46091	53	18.87591	1673.83716	0.7273	Added *Energy*
37	96	0.90330	54	20.77472	1679.08971	0.7278	Added *Strategy*

**Table 9:** LASSO pathways for* Scope 2*

Knot	ID	$\lambda$	*s*	*L*_1_ norm	EBIC	*R*^2^	Action
1	1	3595.51388	1	0.00000	1876.54350	0.0000	Added cons
2	2	3276.09834	2	0.06766	1774.08504	0.0666	Added *Capx*
3	12	1292.16069	3	0.53246	1213.15406	0.3458	Added *Ets*
4	15	977.47218	4	0.75925	1139.48549	0.3783	Added *Operprotio*
5	18	739.42185	6	1.30951	1072.38411	0.4097	Added *Thirty*, *Mtbt*
6	19	673.73366	8	1.51728	1048.47866	0.4379	Added *Managerexe*,* Asset*
7	20	613.88101	10	1.73661	1025.68299	0.4379	Added *Incentive*,* R&D*
8	23	464.37847	11	2.39692	928.14268	0.4737	Added *Inventurn*
9	25	385.53517	12	2.80280	881.88252	0.4913	Added *Leverage*
10	26	351.28529	15	3.05546	875.75194	0.5007	Added *Opexpense510*, *Currentratio*, *Totassgrate*
11	28	291.64322	16	3.63324	824.31350	0.5189	Added *Icp*
12	29	265.73444	18	3.93127	815.31409	0.5330	Added *Risk*, *Carbonmes*
13	30	242.12732	19	4.26317	800.28924	0.5330	Added *Founder*
14	31	220.61740	21	4.58524	796.38700	0.5388	Added *Nationalitymix*,* Sales*
15	32	201.01836	22	4.91012	787.11459	0.5437	Added *Awareness*
16	33	183.16044	24	5.29950	787.63995	0.5481	Added *Finance*, *Receiturntio*
17	34	166.88897	26	5.70959	789.34556	0.5520	Added *CEO*, *Incentiveemp*
18	36	138.55416	29	6.44972	789.94964	0.5585	Added *Dual*, *Value*, *Quickratio*
19	37	126.24539	31	6.82785	795.02853	0.5615	Added *Regulatory*, *Capitalstock*
20	38	115.03009	34	7.26025	808.71957	0.5642	Added *Genderratio*, *Employees*,* ROA*
21	40	95.50000	34	8.06118	791.92995	0.5688	Added *Voluntary*
22	42	79.28578	38	8.80126	810.96844	0.5722	Added *Opexpense*, *Oppo*, *Target*, *Lnlev*
23	46	54.64861	39	10.22887	797.35717	0.5779	Added *Opexpense05*
24	47	49.79378	40	10.53087	801.68230	0.5789	Added* Intensity*
25	48	45.37024	42	10.80744	814.50614	0.5797	Added *Absolute*, *Credit*
26	49	41.33967	44	11.06212	827.76666	0.5804	Added *Touch*, *Totassover*
27	51	34.32092	45	11.51281	831.60719	0.5814	Added *Team*
28	52	31.27195	46	11.71458	837.94778	0.5818	Added* Strategy*
29	55	23.65608	48	12.24798	850.31729	0.5828	Added *Opexpense1015*,* Energy*
30	56	21.55454	49	12.41098	857.32661	0.5830	Added *TDC*
31	57	19.63969	50	12.58872	864.40924	0.5832	Added *Sales*
32	60	14.85671	51	13.09892	870.35433	0.5837	Added *Monetary*
33	63	11.23855	52	13.50541	877.07527	0.5841	Added *Operate*
34	74	4.03893	53	14.33269	883.55202	0.5844	Added *Numberdirectors*
35	85	1.45152	54	14.63054	891.30251	0.5845	Added *Boardamount*

**Table 10:** LASSO pathways for* Scope 3*

Knot	ID	$\lambda$	*s*	*L*_1_ norm	EBIC	*R*^2^	Action
1	1	4218.92733	1	0.00000	2879.83392	0.0000	Added cons
2	2	3844.12945	2	0.10305	2826.68409	0.0484	Added *Capx*
3	9	2004.33228	3	0.68820	2581.42578	0.2252	Added *Ets*
4	10	1826.27293	4	0.84783	2562.33221	0.2420	Added *Thirty*,* Value*
5	13	1381.50849	6	1.52373	2490.20714	0.2897	Added *Strategy*,* Employees*
6	14	1258.77909	7	1.92630	2478.11006	0.3056	Added *Icp*
7	15	1146.95262	8	2.35155	2458.95385	0.3207	Added *Incentive*,* Absolute*
8	16	1045.06051	10	2.79370	2448.73643	0.3350	Added *Genderratio*, *Managerexe*
9	17	952.22022	12	3.44632	2438.28626	0.3490	Added *Regulatory*
10	18	867.62760	13	4.11052	2422.00096	0.3617	Added *Numberdirectors*, *Target*
11	19	790.54995	15	4.70233	2416.51682	0.3727	Added *Energy*, *Voluntary*, *Quickratio*
12	21	656.32846	18	5.78741	2403.35615	0.3912	Added *R&D*
13	22	598.02205	19	6.28535	2394.33381	0.3996	Added *Founder*
14	23	544.89542	21	6.76038	2393.94656	0.4074	Added *Nationalitymix*,* Credit*
15	24	496.48841	23	7.26718	2394.50670	0.4148	Added *Dual*,* Touch*
16	25	452.38175	24	7.74940	2388.98616	0.4211	Added *Lnlev*
17	26	412.19340	26	8.20860	2392.82170	0.4268	Added *Finance*,* Intensity*
18	29	311.80919	31	9.73344	2401.55360	0.4410	Added *Boardamount*, *Sales*, *Operprotio*, *Receiturntio*, *Totassgrate*
19	30	284.10892	34	10.97860	2413.15865	0.4465	Added *Opexpense*, *Opexpense510*, *Carbonmes*, *Capitalstock*
20	38	235.87222	36	13.52114	2407.97526	0.4559	Added *Opexpense05*,* Asset*
21	40	214.91798	37	14.63996	2406.57503	0.4600	Added *TDC*
22	42	178.42868	38	16.56637	2399.81006	0.4664	Added *ROA*
23	46	148.13463	38	18.13149	2389.32461	0.4709	Added *Inventurn*
24	47	122.98397	39	19.44938	2389.83716	0.4741	Added* Team*
25	48	84.76808	40	21.51368	2388.75933	0.4779	Added* Founder*
26	49	77.23752	41	21.96488	2395.14763	0.4786	Added *Oppo*
27	51	70.37595	42	22.37984	2401.77910	0.4791	Added *Target*
28	52	58.42735	44	23.33831	2414.74170	0.4804	Added *Awareness*, *Mtbt*
29	55	48.50741	45	24.20598	2420.41922	0.4813	Added *Incentiveemp*
30	56	44.19815	46	24.61265	2427.41368	0.4817	Added *Opexpense1015*
31	57	36.69408	49	25.34279	2449.72382	0.4824	Added *Operate*, *Currentratio*,* Leverage*
32	60	23.04495	50	26.92072	2455.06221	0.4835	Added *Risk*
33	63	12.01566	51	28.32795	2461.60020	0.4840	Added *Opexpense*
34	74	6.87580	52	29.04216	2469.15565	0.4842	Added *Monetary*
35	85	2.97637	54	29.60838	2484.87427	0.4843	Added *Totassover*,* CEO*

**Table 11:** LASSO pathways for *Rin*

Knot	ID	$\lambda$	*s*	*L*_1_ norm	EBIC	*R*^2^	Action
1	1	2531.85816	1	0.00000	2208.61323	0.0000	Added cons
2	2	2306.93486	4	0.18055	2152.01180	0.0477	Added *Opexpense05*, *Currentratio*, *Capx*
3	4	1915.25785	5	0.55509	1996.98019	0.1375	Added *R&D*
4	6	1590.08071	6	0.86226	1862.44383	0.2091	Added *Totassover*
5	8	1320.11294	12	1.23446	1779.97143	0.2692	Added *Operate*, *Icp*, *Thirty*, *Ets*, *Touch*,* Asset*
6	10	1095.98096	14	1.81794	1644.88801	0.3333	Added *Risk*, *Mtbt*
7	12	909.90265	15	2.36935	1504.03921	0.3910	Added* Leverage*
8	14	755.41716	17	2.87367	1397.39906	0.4347	Added *TDC*, *Quickratio*
9	15	688.30798	19	3.18919	1350.02123	0.4560	Added *Target*,* Incentive*
10	20	432.27747	20	4.46868	1144.48571	0.5222	Added* Regulatory*
11	21	393.87513	22	4.78791	1130.58511	0.5308	Added *Opexpense*, *Genderratio*
12	23	327.00206	24	5.52347	1096.27010	0.5449	Added *Sales*, *Operprotio*
13	24	297.95209	26	5.99783	1089.50310	0.5511	Added *Team*, *Dual*
14	25	271.48285	27	6.45077	1076.91770	0.5567	Added *Employees*
15	26	247.36506	28	6.86775	1067.55539	0.5613	Added *Carbonmes*
16	27	225.38983	30	7.28831	1067.41439	0.5656	Added *CEO*, *Inventurn*
17	28	205.36682	31	7.70903	1060.00663	0.5696	Added *Incentiveemp*
18	29	187.12260	32	8.13678	1053.19740	0.5734	Added *Opexpense510*
19	30	170.49914	33	8.54920	1047.75285	0.5769	Added *Totassgrate*
20	32	141.55138	36	9.38635	1047.26554	0.5831	Added *Finance*, *Absolute*,* ROA*
21	33	128.97635	38	9.85912	1051.04359	0.5862	Added *Opexpense05*,* Energy*
22	34	117.51845	38	10.34864	1039.78391	0.5890	Added *Boardamount*
23	35	107.07843	40	10.87442	1045.88087	0.5914	Added *Receiturntio*, *Capitalstock*
24	36	122.98397	41	11.42561	1045.27664	0.5935	Added* Incentive*
25	37	84.76808	42	11.92671	1045.98136	0.5953	Added *Numberdirectors*
26	40	67.24838	43	13.18474	1038.00792	0.5992	Added *Oppo*
27	41	61.27421	47	13.55800	1065.69877	0.6002	Added *Strategy*, *Managerexe*, *Voluntary*,* Value*
28	42	55.83078	48	13.91802	1069.89623	0.6011	Added* Lev*
29	43	50.87093	49	14.24890	1074.67661	0.6019	Added* Credit*
30	47	35.06336	50	15.36092	1073.99245	0.6040	Added *Asset*
31	48	31.94843	51	15.62925	1080.23017	0.6044	Added *Nationalitymix*
32	49	29.11022	52	15.88238	1086.71150	0.6047	Added* Monetary*
33	63	26.52414	53	16.12761	1093.33618	0.6050	Added* Awareness*
34	74	4.97014	54	18.18020	1095.01917	0.6065	Added *Founder*

**Table 12:** LASSO pathways for *Ein*

Knot	ID	$\lambda$	*s*	*L*_1_ norm	EBIC	R^2^	Action
1	1	4017.58140	1	0.00000	2539.67894	0.0000	Added cons
2	2	3660.67054	2	0.09734	2455.72948	0.0543	Added* Asset*
3	5	2769.16302	3	0.34115	2252.19551	0.1684	Added Capx
4	7	2299.00779	4	0.61105	2083.95141	0.2529	Added *Opexpense05*
5	8	2094.77027	5	0.78460	2003.99018	0.2917	Added *Currentratio*
6	9	1908.67665	6	0.96409	1912.11267	0.3334	Added* R&D*
7	11	1584.61689	7	1.31836	1746.46010	0.4002	Added *Operprotio*
8	12	1443.84389	8	1.66254	1665.95348	0.4316	Added *Ets*
9	13	1315.57678	9	2.00318	1587.97659	0.4605	Added *Touch*
10	14	1198.70457	11	2.34149	1521.57404	0.4868	Added *Icp*,* Thirty*
11	16	995.18559	13	3.02887	1387.58619	0.5315	Added *Operate*,* Risk*
12	18	826.22056	14	3.64173	1271.88683	0.5655	Added *Mtbt*
13	19	752.82140	15	3.87744	1221.22659	0.5807	Added* TDC*
14	21	625.00555	16	4.30186	1128.80170	0.6055	Added* Incentive*
15	22	569.48178	18	4.59783	1093.28183	0.6176	Added *Quickratio*,* Leverage*
16	27	357.65116	20	5.86543	938.04154	0.6554	Added *Team*, *Carbonmes*
17	28	325.87841	23	6.17616	936.90494	0.6606	Added *Opexpense*, *Genderratio*
18	29	296.92827	24	6.54167	921.45495	0.6654	Added* Regulatory*
19	30	270.54998	26	6.90073	915.71689	0.6697	Added *Dual*, *Inventurn*
20	31	246.51507	29	7.25779	919.11597	0.6738	Added* Employees*, *Target*, *Incentiveemp*
21	33	204.66114	31	7.93329	899.91153	0.6807	Added *CEO*,* Incentive*
22	34	186.47961	32	8.23725	893.69335	0.6834	Added *Totassgrate*
23	37	141.06499	35	9.03862	886.19316	0.6894	Added *Boardamount*, *Opexpense510*,* Absolute*
24	38	128.53316	36	9.42033	883.02381	0.6915	Added* Opexpense1015*
25	39	117.11463	37	9.81113	880.52760	0.6935	Added* Finance*
26	40	106.71049	38	10.20201	878.74167	0.6953	Added *Numberdirectors*
27	41	97.23062	40	10.55641	886.03117	0.6968	Added *Monetary*,* Energy*
28	42	88.59292	41	10.88559	886.59444	0.6982	Added* Voluntary*
29	44	73.55140	46	11.55877	913.91115	0.7005	Added *Strategy*, *Nationalitymix*, *Credit*, *Capitalstock*
30	47	55.63894	47	12.60574	907.80676	0.7030	Added *Sales*
31	51	38.34976	48	13.69392	905.50937	0.7049	Added *Managerexe*
32	53	31.83865	49	14.11261	910.29160	0.7054	Added *Totassover*
33	58	19.99560	50	14.86886	913.78089	0.7062	Added* Founder*
34	60	16.60070	51	15.09315	920.80387	0.7064	Added *Value*
35	63	12.55782	52	15.36587	927.85883	0.7065	Added *Receiturntio*
36	69	7.18605	53	15.78801	934.96543	0.7067	Added* ROA*
37	76	3.74681	54	16.10655	942.55500	0.7068	Added* Awareness*
